# MINErosion 3: Using measurements on a tilting flume-rainfall simulator facility to predict erosion rates from post-mining landscapes in Central Queensland, Australia

**DOI:** 10.1371/journal.pone.0194230

**Published:** 2018-03-28

**Authors:** Hwat Bing So, Ashraf M. Khalifa, Bofu Yu, Chris Caroll, Peter Burger, David Mulligan

**Affiliations:** 1 School of Engineering and Built Environment, Australian Rivers Institute, Griffith University, Nathan, Queensland, Australia; 2 School of Land and Food Sciences, The University of Queensland, St Lucia, Queensland, Australia; 3 Department of Natural Resources, Mines and Energy, Rockhampton, Queensland, Australia; 4 Centre for Mined Land Rehabilitation, Sustainable Minerals Institute, The University of Queensland, St Lucia, Queensland, Australia; Pennsylvania State University University Park: Penn State, UNITED STATES

## Abstract

Open-cut coal mining in Queensland results in the formation of extensive saline overburden spoil-piles with steep slopes at the angle of repose (approximately 75% or 37^o^). These spoil-piles are generally found in multiple rows, several kilometers in length and heights of up to 50 or 60 m above the original landscape. They are highly dispersive and erodible. Legislation demands that these spoil piles be rehabilitated to minimize on-site and off-site discharges of sediment and salt into the surrounding environment. To achieve this, the steep slopes must be reduced, stabilized against erosion, covered with topsoil and re-vegetated. Key design criteria (slope gradient, slope length and vegetation cover) are required for the construction of post-mining landscapes that will result in acceptable erosion rates.

A novel user-friendly hillslope computer model MINErosion 3.4 was developed that can accurately predict potential erosion rates from field scale hillslopes using parameters measured with a 3m laboratory tilting flume-rainfall simulator or using routinely measured soil physical and chemical properties. This model links MINErosion 2 with a novel consolidation and above ground vegetation cover factors, to the RUSLE and MUSLE equations to predict the mean annual and storm event erosion rates. The RUSLE-based prediction of the mean annual erosion rates allow minesites to derive the key design criteria of slope length, slope gradient and vegetation cover that would lead to acceptable erosion rates. The MUSLE-based prediction of storm event erosion rates will be useful as input into risk analysis of potential damage from erosion.

MINErosion 3.4 was validated against erosion measured on 20 m field erosion plots established on post-mining landscapes at the Oakey Creek and Curragh coalmines, as well as on 120 and 70 m erosion plots on postmining landscapes at Kidston Gold Mine.

## Introduction

**Fig 1:**
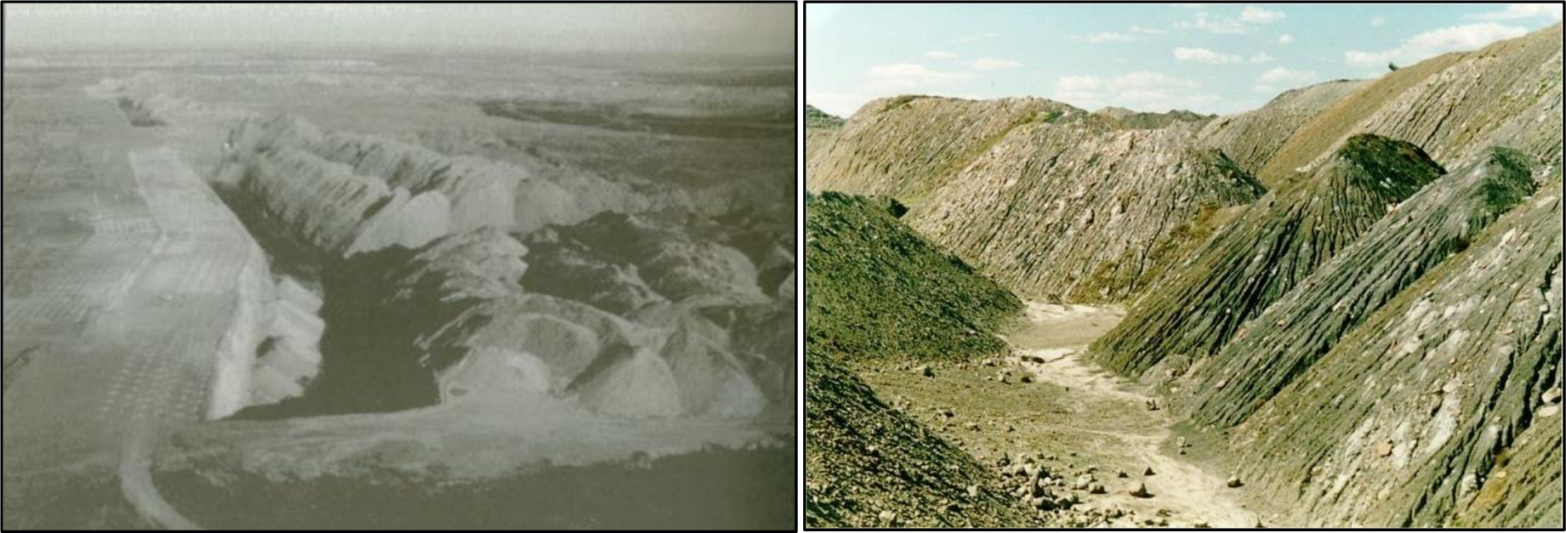
On the left is a typical long parallel overburden spoil piles produced by dragline operation in a Central Queensland open cut coal mine[2] and a close-up view of the spoil-piles that can reach 50 to 60 m high [1]. Note that the working pit (left photo) is flanked by the high wall on one side and fresh spoil piles on the other side.

The nature of open-cut mining is that much larger surface area is disturbed compared to underground mining. In Australia, the deep solid overburden above the mineral or coal seam is blasted using explosives and then removed mechanically using trucks & shovels or draglines. The latter is the most common method used in Central Queensland open-cut coal mines. The high speed of operation using draglines results in landscapes that consist of long parallel tertiary overburden spoil-piles that are generally highly saline, dispersive and highly erodible as shown in Fig 1 [1, 2]. The height of these spoil-piles may exceed 50–60 m above the original landscape and the slopes are at the angle of repose of around 75% or 37° [3]. Legislation and public opinion require that these highly disturbed open-cut post-mining landscapes be satisfactorily rehabilitated for approved post-mining land use.

In Central Queensland, where most of these open-cut mines are located, annual rainfall may range from 200 mm to 1400 mm, with a long-term median of around 600 mm and dominated by several intense tropical rainstorms annually which may reach intensities of over 100 mm.hr^-1^. These storms will result in severe erosion unless the slope length and steepness are reduced and the disturbed land rehabilitated and revegetated satisfactorily.

In Queensland alone, the total area of land disturbed by open-cut coal mining and land already rehabilitated exceeds 146,000 and 44,000 hectares, respectively [4]. Under current legislation, mines must apply for an Environmental Authority (EA) before mining operation can be conducted and a Financial Assurance (FA) may be required as part of the EA. FA is a type of security provided to the Government if a company fails to adequately rehabilitate the disturbed landscape. It is based on third party costing as the government may have to employ contractors (third party) to conduct the rehabilitation activity should the mine fail to meet its obligation to rehabilitate the site satisfactorily [5]. It is the responsibility of the company to develop an appropriate estimation of the FA based on the DEHP Financial Assurance Calculator which has a detailed list of default unit costing for each step of the rehabilitation operation.

The most expensive component of the rehabilitation process is the re-shaping and preparation of the overburden dumps to create a suitable landscape for vegetation growth. Landscape reconstruction typically involves extensive and costly earthworks to produce a post-mining landscape that is resistant to geo-technical failure and surface erosion processes by rainfall and runoff. In the case of high risk overburden material (e.g. pyritic material that must be buried) the DEHP Financial Assurance Calculator provided estimates of default third party cost for reshaping and capping as $136,000 per ha [5].

Severe erosion can occur on post-mining landscapes if slopes are too steep and may result in rehabilitation failures, excessive sediment and salt discharges onto surrounding areas and waterways as well as the mining operational areas which could result in additional costly interruption of mine operations. As the marginal cost per additional degree of slope reduction increase progressively as the final slope gradient is reduced (associated with greater earthworks per degree of slope reduction), and as soil and overburden materials exhibit a very wide range of erodibilities [1], the extent and cost of earthworks can be minimised, and rehabilitation failures avoided, if soil erosion from design landscapes can be predicted prior to construction.

A methodology that assist mine-sites to efficiently and economically design post-mining landscapes prior to reconstruction, and that will result in acceptable erosion rates (average annual erosion rates less than or equal to the natural erosion rates of the surrounding area) would be useful. As these landscapes are particularly vulnerable to erosion during the first few years following rehabilitation and prior to the establishment of adequate vegetation cover, this methodology should also allow the prediction of the probability of damaging erosion events from rainstorms.

Existing erosion models such as the Universal Soil Loss Equation (USLE) [6], and its revised version RUSLE [7], the Water Erosion Prediction Project (WEPP) [8] were developed mainly for agricultural conditions where the topography is given, and modifying the slope gradient is not an option, and where soils are cultivated and disturbed seasonally or annually. Therefore, soils are subjected to only limited seasonal consolidation (increase in bulk density and strength) between cultivation and are often cleared of rocks where they impede soil cultivation. Crops are sown onto cultivated land and they progressively develop their canopy and root systems, reaching a maximum at around flowering and then decline towards the end of the season. The erosion models generally require input data collected using field erosion plots conducted on the soil of interest and crops grown on them, which are resource intensive and time consuming. Soil erodibility used in these models refers to the property of the freshly cultivated land, and as the crop develops, the vegetation cover factor includes the effect of roots in consolidating the soil.

In contrast, in the management of post-mining landscapes, changing slope gradients and slope lengths are the essential first steps necessary to stabilize the landscape. Overburdens are often high in rock content which provides protection from water erosion, while top-soils are often limited in quantity, of poor fertility and may consist of coarse grained materials such as fine gravel. The surface soil or overburden are vegetated, left undisturbed and allowed to consolidate (increase in bulk density and strength) with time. Vegetation cover fluctuates seasonally and may disappear temporarily due to the bushfire which is a natural part of the Australian ecology. In the latter situation, the soil has been consolidated by the processes of wetting and drying and the presence of roots. Therefore, separating the effect of above ground vegetation and the consolidation of the soil including the roots would allow the estimation of erosion rates from bare soils following bushfires. Existing agriculture based erosion models were not intended to design new landscapes for erosion control on post-mining landscapes and a new approach was needed for that purpose.

**Fig 2:**
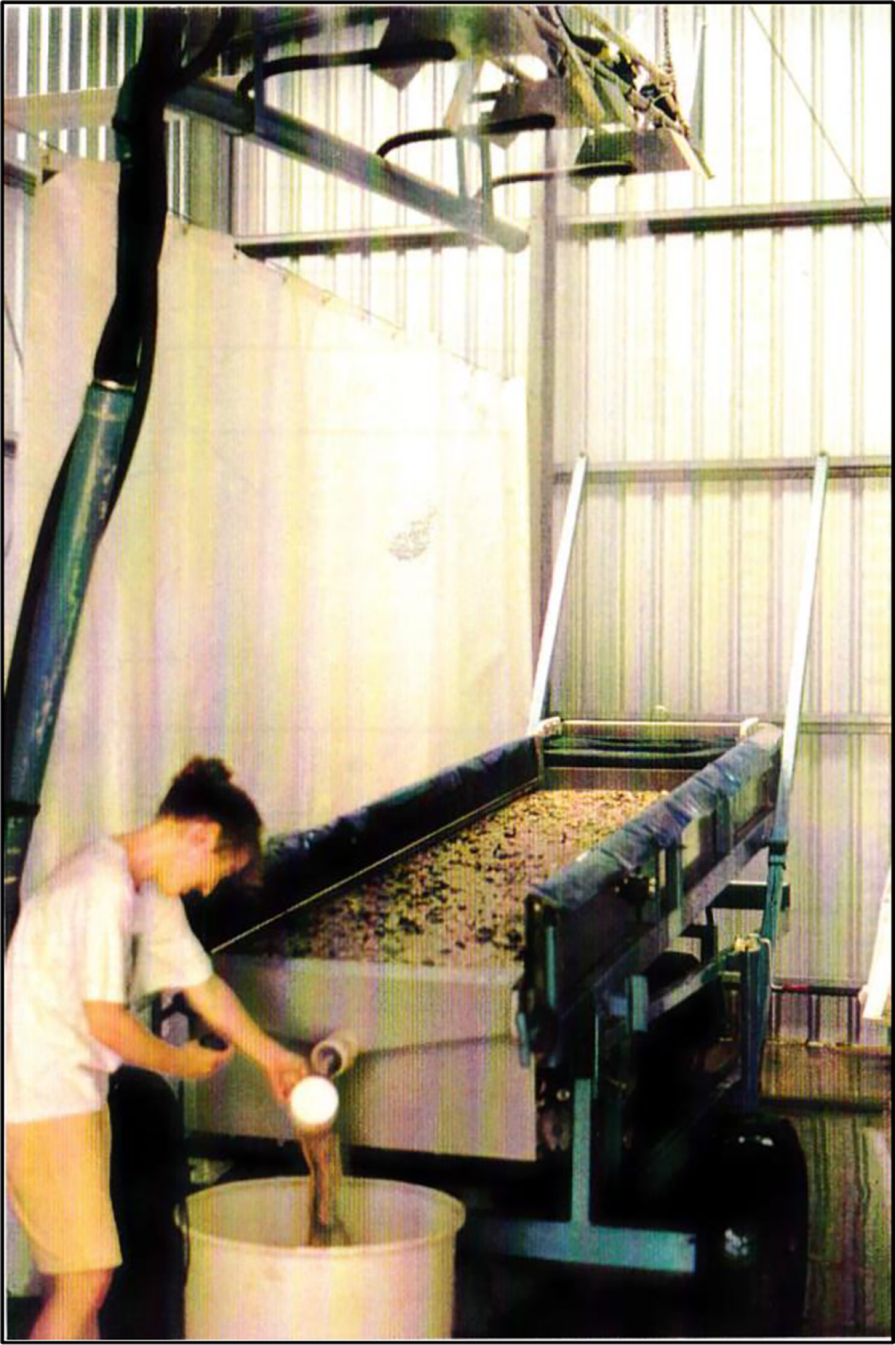
Measuring run-off and sediment concentration under the laboratory rainfall simulator-tilting flume facility.

The MINErosion 2 model was developed to overcome the problem of scaling across different length of hillslopes. It combines the separate laboratory measured rill and inter-rill erodibility parameters into a combined erodibility and the prediction of erosion from unconsolidated (freshly applied) material at the hillslope scale of Central Queensland [1] The concept that underpins MINErosion 2 is that erosion is dominated by a few intense rainstorm events where run-off quickly reached steady state conditions, which is characteristic of tropical rainfall in Central and Northern Queensland. These rainstorms represent the worst-case scenarios that are of greatest relevance in the design of post-mining landscapes because of their potential for damage. Rill and inter-rill erodibilities and slope adjustment factors were measured for thirty-four soil/overburden materials from Central Queensland coalmines on a tilting flume (3 m long x 0.8 m wide, slope adjustable from 0 to 50%) at the University of Queensland Erosion Processes Laboratory as shown in Fig 2 [1]. Each material was subjected to 100 mm.h^-1^ rainstorm for 30 minutes (equivalent to a 1-in-20-year event in Central Queensland) at 20% slope, followed by slopes of 5, 10, 15 and 30% for 15 minutes each. At these rainfall intensities, steady state was quickly established. The data from these measurements and the derived parameters were used to develop the MINErosion 2 model, which successfully estimates field scale erosion rates on bare unconsolidated simple linear hillslopes with various combinations of slope gradients and lengths. MINErosion 2 can also be used effectively to simulate multiple field plot experiments on a computer, based on a few measurements made on a tilting flume-rainfall simulator facility in the laboratory. This is a highly efficient experimental procedure.

The model was developed as an Excel spreadsheet, with a user interface written in MS Visual Basic [9]. Erodibility parameters and other soil properties for 34 different soil and overburden types from Central Queensland open-cut coalmines were embedded as a dataset in the model and details of this dataset and the methodology used to measure them can be found in an earlier paper [10]. The relationship between rill/inter-rill erodibility and relevant soil properties were also embedded in the model, and where erodibility values of a new site is unknown, the model provides an option to estimate the rill and inter-rill erodibilities from routinely measured soil properties. The equations that underpin MINErosion 2 have been published [1, 11]

The main output from MINErosion 2 includes rill, inter-rill and total soil loss from different combinations of slope and slope length on hillslopes with bare unconsolidated soil or overburden [11]. However, MINErosion 2 cannot be used to predict erosion from field plots that has been vegetated and left to consolidate for some years and require further development which culminated in the MINErosion 3 model.

Mine-sites require assessments of the potential annual erosion rates from rehabilitated areas for their environmental reporting and completion criteria reporting to the DEHP (Department of Environment and Heritage Protection), the authority responsible for rehabilitation monitoring and compliance. In addition, mine-sites are also concerned about the risk of erosion damage to the rehabilitated landscapes during the early years prior to the establishment of vegetation cover. In the transition period from bare unconsolidated to vegetated and consolidated soil/spoil, appropriate algorithms and models for the effect of above-ground vegetation cover and consolidation had to be developed.

As the RUSLE mean annual erosion rates are required by DEHP as a measure of erosion rates, MINErosion 2 was then linked to the RUSLE equations by incorporating the above ground vegetation cover and consolidation factors, to predict mean annual erosion rates, and to the Modified USLE or MUSLE [12] equation to predict rainstorm event erosion rates. These linkages are developed in this paper as part of MINErosion 3.

Therefore, the objectives of this paper are:

1. To develop the above-ground vegetation cover (C) and consolidation (Ccons) factors that are necessary to apply RUSLE and MUSLE to rehabilitated mine-sites;
2. To develop MINErosion 3 as a tool for the prediction of event-based and annual soil erosion rates from soil erodibility measured with a tilting flume-rainfall simulator facility in the laboratory or from routinely measured soil physical and chemical properties;
3. To validate the MINErosion 3 model with field data collected using erosion plots in the field.

## Materials and methods

### Development of the vegetation cover and consolidation functions

The **vegetation cover (C)** function was developed from measurements on the tilting flume-rainfall simulator facility shown in Fig 2 [13]. This function defines the factor that reduces the erosion rate in response to changes in the above-ground vegetation cover. It replaces the vegetation cover factor in the RUSLE equation.

The tilting flume was equipped with 8 inserts (3 m long x 0.8 m wide x 0.25m deep), which can be lifted into the flume for a simulation run and lifted out and placed in the glasshouse. The inserts were packed with 2 soil types, a heavy black clay (81% clay, 11% silt, 8% sand) and a sandy loam (21% clay, 29% silt, 50% sand)), planted with 2 types of grasses (a tussocky Rhodes grass, *Chloris guyana*, and a stoloniferous Sabi grass, *Urochloa mosambicensis*) and 2 replications. Plants were watered regularly and maintained at the field capacity. After reaching adequate growth with more than 50% cover, they were trimmed successively to have 50%, 25%, 10% and 0% standing vegetative cover. Trimming was conducted immediately following the completion of a series of measurements at each level of cover and each series is completed within one or two days. Visual and photographic assessments were made of the vegetative cover. At each stage, 30 minutes’ rainfall simulations (under 100 mm.hr^-1^ rainfall) were conducted at 10, 15, 20 and 30% slope. Samples of run-off were collected for 10 seconds each at 3, 5, 8, 12, 16, 20 and 25 minutes, to determine run-off rates and sediment concentrations. As the steady state is achieved within 15 mins, the average of the last 3 measurements were used as the steady state run-off and sediment concentrations. The results were statistically analysed by computing the standard errors of the mean values, and an analysis of variance conducted at the p = 0.05 to determine the effect of soil, slope and vegetation cover on steady state run-off, steady state sediment concentration, soil loss and relative soil loss rates. Details of this procedure are given in S1 File.

Total event soil loss, steady state run-off and soil loss rates were calculated. As soil consolidation levels remain the same, the relative steady state soil loss rates (vegetated/bare) were calculated for each level of vegetative cover to develop the vegetation cover function independent of soil properties.

The reduction of the above-ground vegetation cover was also intended to simulate the effect of frequent loss of cover by fire (which is a regular feature of Australia’s native ecosystem) which leaves roots intact within the soil. The effect of roots in the soil was included as part of the soil consolidation factor (see below). The separation of roots from the above ground component is different from the USLE models [14] where vegetation cover includes its root system. It is a simplification of the vegetation cover function from RUSLE and RUSLE2 where the vegetation cover function is made up of several sub-factors [15]. The root function is one such factor.

The **consolidation function (C**_**cons**_**)** was derived from field measurements of soil erodibilities on two mine-sites and described in details in S2 File This function defines the reduction of K_MUSLE_ associated with the age of rehabilitation. At the Blair Athol Mine, Central Queensland (22^o^41’S,147^o^31’E) measurements were taken on a sandy loam (yellow Podzolic or duplex alfisol, 24% clay) and clay soils (Black Earth or vertisol, 55–57% clay) using a 4-m long field rainfall simulator similar to that shown in Fig 2, which has the same design as the laboratory simulators [16, 17]. For each soil type, a range of consolidation or rehabilitation ages were selected. For the sandy loam, the age ranged from 0 (freshly rehabilitated), 4 and 15 years of consolidation and an unmined and undisturbed site (nominally represented as 30 years) and on the clay soil type, age range from 0, 2 and 15 years of consolidation and an unmined site (nominally represented as 30 years). Sites were selected for similar slope gradients with a range from 3 to 8%. For each case, three replicate plots were prepared and averaged and the standard error calculated.

Similar simulations were also conducted at the Mount Isa Mines, Queensland (20^o^ 43’ S, 139^o^ 29’ E) on a sandy loam (red duplex alfisol with 14–22% clay) with some rocks and gravel [18]. Age of rehabilitations were 0, 3 and 7 years of consolidation and an undisturbed and unmined site (nominally represented as 30 years). For each case, three replication plots were prepared and averaged and a standard error calculated.

All above ground vegetation was removed and rainfall simulation was conducted over a plot of 4 m x 1.5 m using 100 mm.hr^-1^ for 30 minutes to measure inter-rill erodibilities, followed by a series of consecutive overland flow with discharge rates between 0.5 to 3 L.s^-1^ (as run-on water) to measure rill erodibilities. Run-off water and sediment were measured at different intervals during the simulations. Total sediment delivery, steady state run-off and sediment delivery rates were calculated from the data, and used to calculate rill and inter-rill erodibilities of the sites. Relative K_MUSLE_ (Consolidated/Unconsolidated erodibilities) were calculated and plotted against the age of rehabilitation to develop the consolidation function. As roots were left in the soil and considered as part of the soil, its effect was included in the consolidation factor.

For each site, bulk soil samples were collected with a backhoe and transported to the laboratory for a standard simulation on the tilting flume to determine the slope adjustment factors for each soil material. For each flume, three 200 l drums of soil are required and are sieved to <50 mm before use.

### The MINErosion 3 framework

The starting point for MINErosion 3 is the MINErosion 2 model, and details of the equations involved have been published previously [1, 11], and included in S3 File for interested readers.

The output from MINErosion 2 includes the rill, inter-rill and total erosion rates per 1m plot width for any selected slope and slope length combinations of bare unconsolidated soil/spoil on simple uniform slopes [19]. For prediction of mean annual erosion rates with RUSLE, and event erosion rates from rainstorms other than 100 mm.hr^-1^ and on slopes that have been vegetated and left to consolidate with time, MINErosion 2 needs to be developed further and linked to the RUSLE and MUSLE equations.

In MINErosion 3, the rill and inter-rill erodibilities from MINErosion 2 are combined with estimates of the total runoff volume (Q) and peak runoff rate (q_p_) into a a single-valued erodibility using the MUSLE equation. This was done by simulating erosion for a standard plot (22.1 m long, slope of 9%) on bare unconsolidated soil/spoil to derive the erodibility K_MUSLE_ as defined by the MUSLE equation [12]

$$A=(0.5\;{EI}_{30}+0.349\;Qq_{p}^{0.333})\;K_{MUSLE}\;LS\;C\;P$$ (1)

where

- EI_30_ is the erosivity index of the simulated rainstorm, calculated by the following equation [20]:

$$•\;{EI}_{30}=[11.9+8.7log(I)]I^{2}D/100$$ (2)

- Q and q_p_ are the total runoff (mm) and the peak runoff rate (mm.hr^-1^), respectively. At the steady state, they are estimated for the simulated conditions from the following equations
  $$Q=(I-I_{r})D$$ (3)
  and q_p_ is the peak run-off rate(mm.hr^-1^), *I* is the rainfall intensity (mm.hr^-1^), *I*_r_ is the steady state infiltration rate (mm.hr^-1^) and D is the rainfall event duration in hr.
- LS is the USLE slope length and gradient factor for the standard plot, and C and P are the above-ground vegetation cover factor described above and soil protection factors (both assumed as 1.0 in Eq (1) for the calculation of K_MUSLE_).

When MINErosion 3 is applied to vegetated and consolidated soil/spoil, the soil erodibility K_MUSLE_ is modified by the empirically determined consolidation factor. Fundamentally, the soils susceptibility to erosion or the erodibility factor should be the same for both USLE/RUSLE and MUSLE equations. The difference between the two equations is in the erosivity values used [12]. Therefore, in MINErosion 3 K_MUSLE_ is used for both the MUSLE and RUSLE equations.

For the estimation of event-based erosion rates, a relationship between the recurrence interval (probability of occurrence) of rainstorm intensity and the rate of erosion is generated. For any given rainfall intensity above 60 mm.hr^1^ (100 mm.hr^-1^ as default value), storm duration and its recurrence interval can be estimated using the IFD (intensity-frequency-duration) curve for the location [21, 22]. Storm EI30 is calculated for the storm of a given intensity, duration and recurrence interval. Soil loss for that event is estimated with the relevant L and S factors using the MUSLE equation. This calculation is repeated using various rainfall intensities and durations and the resulting erosion rates plotted against recurrence intervals. The default calculation is done for a bare, unconsolidated soil/spoil under 100 mm/h rainstorm intensity and repeated for selected periods of consolidation and degree of vegetation cover. The output is presented as graphs and can be printed as either graphs or tables, or both.

The mean annual soil loss was computed using the RUSLE, and a relationship between the average annual soil loss rate and slope length is generated for various slope gradients. The mean annual EI_30_ is computed using the long-term average rainfall pattern for the selected location (default is Emerald, Central Queensland). The default calculation is for a bare, unconsolidated soil/spoil. Computation is repeated for soil/spoil that has been consolidated for various periods and degrees of vegetation cover for selected combinations of slope lengths and slope gradients. Mean annual soil loss rates are then plotted against slope length for selected slope gradients. As the slope gradient, slope length and vegetation cover are the three landscape factors relevant for design and construction of post-mining landscapes that will result in acceptable (annual) erosion rates, these graphs can readily be used to derive suitable landscape design factors.

**Fig 3:**
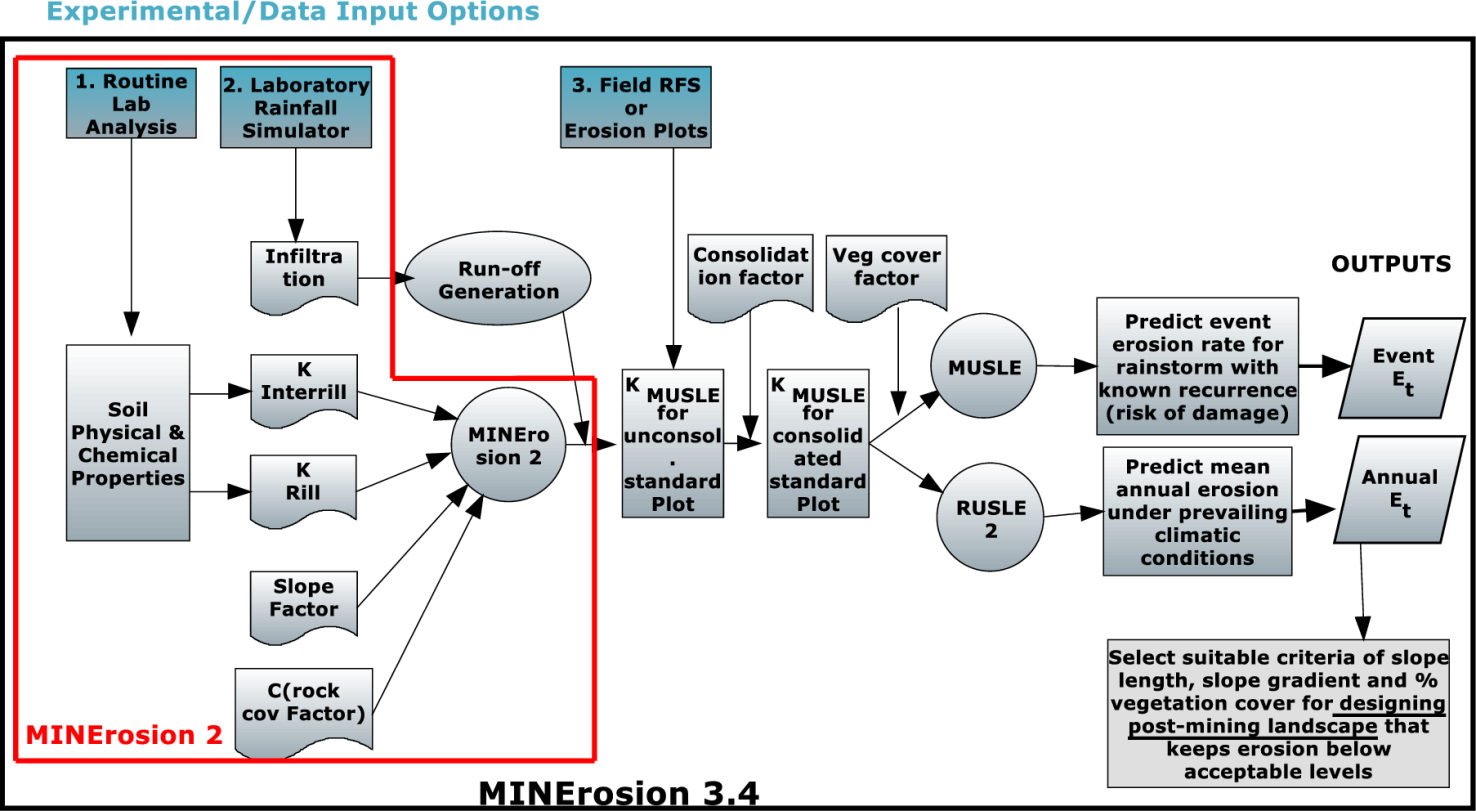
The flowchart of MINErosion 2 and 3.4 models.

The flow chart and the processing steps of the MINErosion 2 and 3 models are presented in Fig 3.

### Field data collection from 3 experimental sites for validation

**Fig 4:**
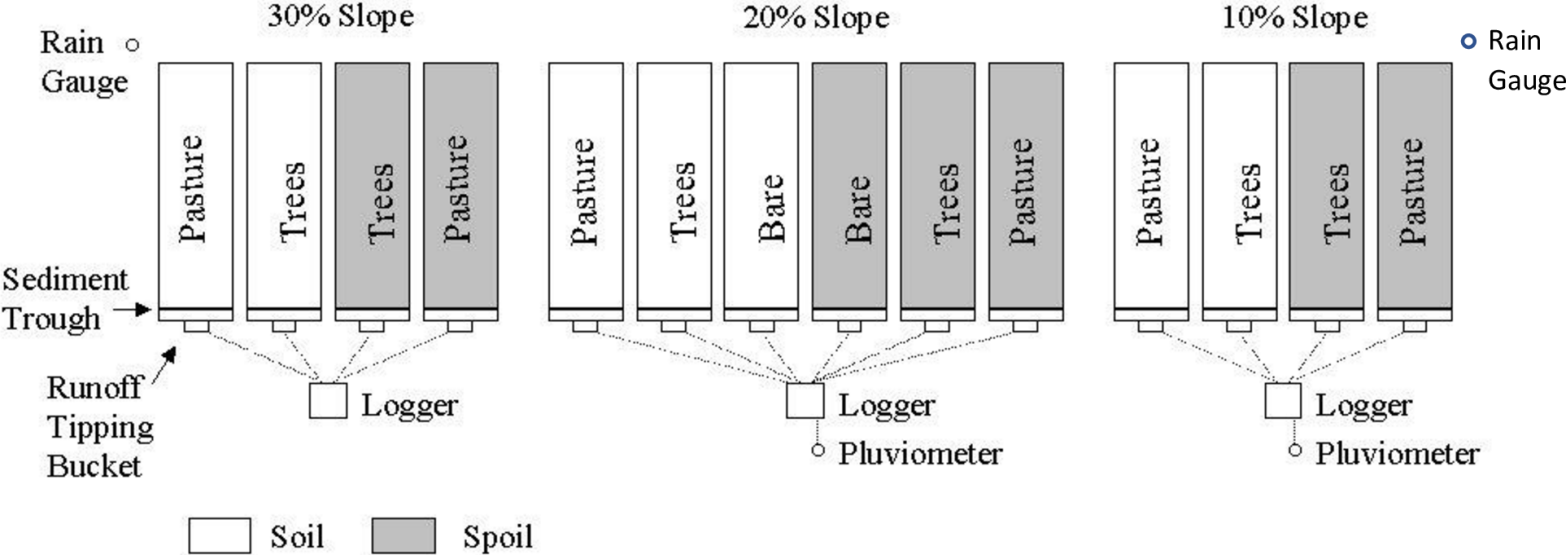
The experimental plots at Curragh mine [20].

Field erosion plots were initiated on the Curragh (23^o^ 30’ S, 148^o^ 50’ E) and Oaky Creek mines (23^o^4’ S, 148^o^ 28’ E) near Emerald, Queensland in 1992. These field plots were 20 meters long (up and down the slope) and 5 meters wide across the slope. The slope varied from 10, 20 and 30% with different percentage of vegetation cover of trees and grasses and time of consolidation [23], however the trees were not well established and were predominantly covered with grass. There were a total 14 plots at each site as shown in Fig 4. The soil plots consist of 20–30 cm topsoil over spoil and plots were bounded to isolate run-on from the top and sides.

The site was instrumented with 2 recording rain-gauges at the top of the 10% and 30% plots. Each plot had a double bedload sediment trough at the bottom to collect the coarse sediment (bedload). The total run-off volume and peak run-off rate were recorded with a tipping bucket and the number of tip per minute was recorded on a data-logger. Total tips were also recorded on a mechanical counter as a backup. To determine suspended sediment concentration in the run-off, a small sample (0.1% of flow) was collected using a 10 cm copper tube with a 1 mm slit and accumulated in a 20 l plastic drum. A sub-sample was taken to determine suspended solid concentration, pH and EC. Soil loss was measured as the sum of bedload and suspended sediment load. Bedload was collected after each event and weighed. Vegetative cover was quantified from 3 photographs taken at 5 m intervals and covered an area of 30 m^2^, and the standing dry matter was determined in year 6 (1998) on the soil plots by cutting and drying.

**Fig 5:**
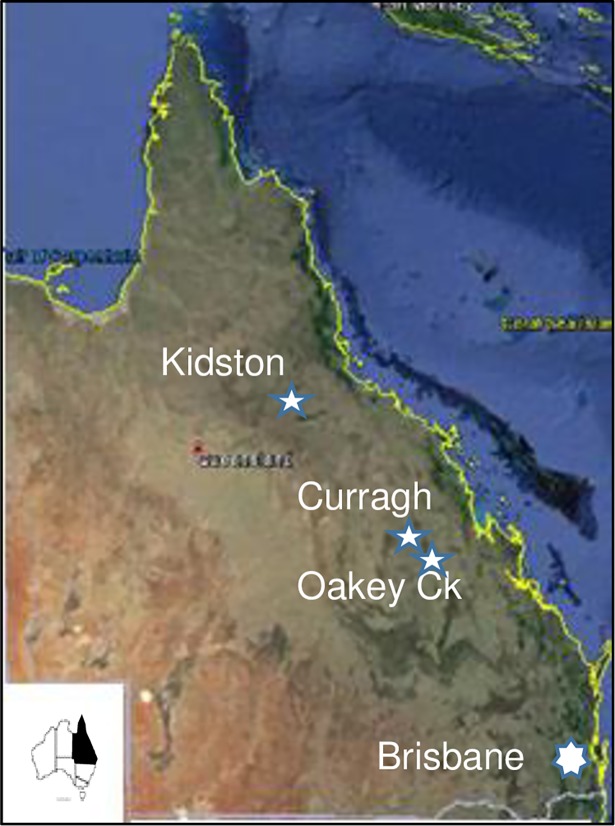
Map showing the location of the three mines in Central Queensland, Australia.

The location of the three sites is shown in Fig 5. The third site was at Kidston Goldmine (19^o^ 6’ S, 143^o^ 26’ E) where in 1996 erosion plots of 120 m long and 20 m wide were setup on 20^o^ (44%) slopes, and 70 m long plots x 20 m wide on 37^o^ (75%) slopes [24]. There were three treatments (spoil + veg; spoil + soil—veg; spoil + soil + veg) x 2 replications on the 20^o^ slopes, and two treatments (spoil + veg; spoil + soil + veg) x 2 replications on the 37^o^ slopes. There were also two older plots initiated in 1991 on 17^o^ and 37^o^ consisting of spoil + vegetation treatments. Soil capping consists of 20–30 cm soil material. Run-off and soil loss were measured in a similar fashion as at the other two mine-sites. Because of the size of the plots, a 3-trough system was used to collect and measure the bedload. This comprises a modified triple Gerlach troughs (20m x 0.5m x 0.4m each), to collect soil and water and direct the water either through simple manifolds to tipping buckets, which measure total flows and thence to automatic samplers for collection of samples for sediment analysis or through modified Parshall flumes, which measure flow rates and total water volume and thence to automatic samplers. Five automatic samplers are being used on plots where high runoff of water and sediment is expected on the 20^o^ slopes and five tipping buckets in lower runoff situations on the 37^o^ slopes.

Details of the procedures used for validation of annual and individual rainstorm event erosion rates are given in S3 File.

## Results

### The vegetation cover (C) and consolidation (C_cons_) factors

#### Vegetation cover C

**Fig 6:**
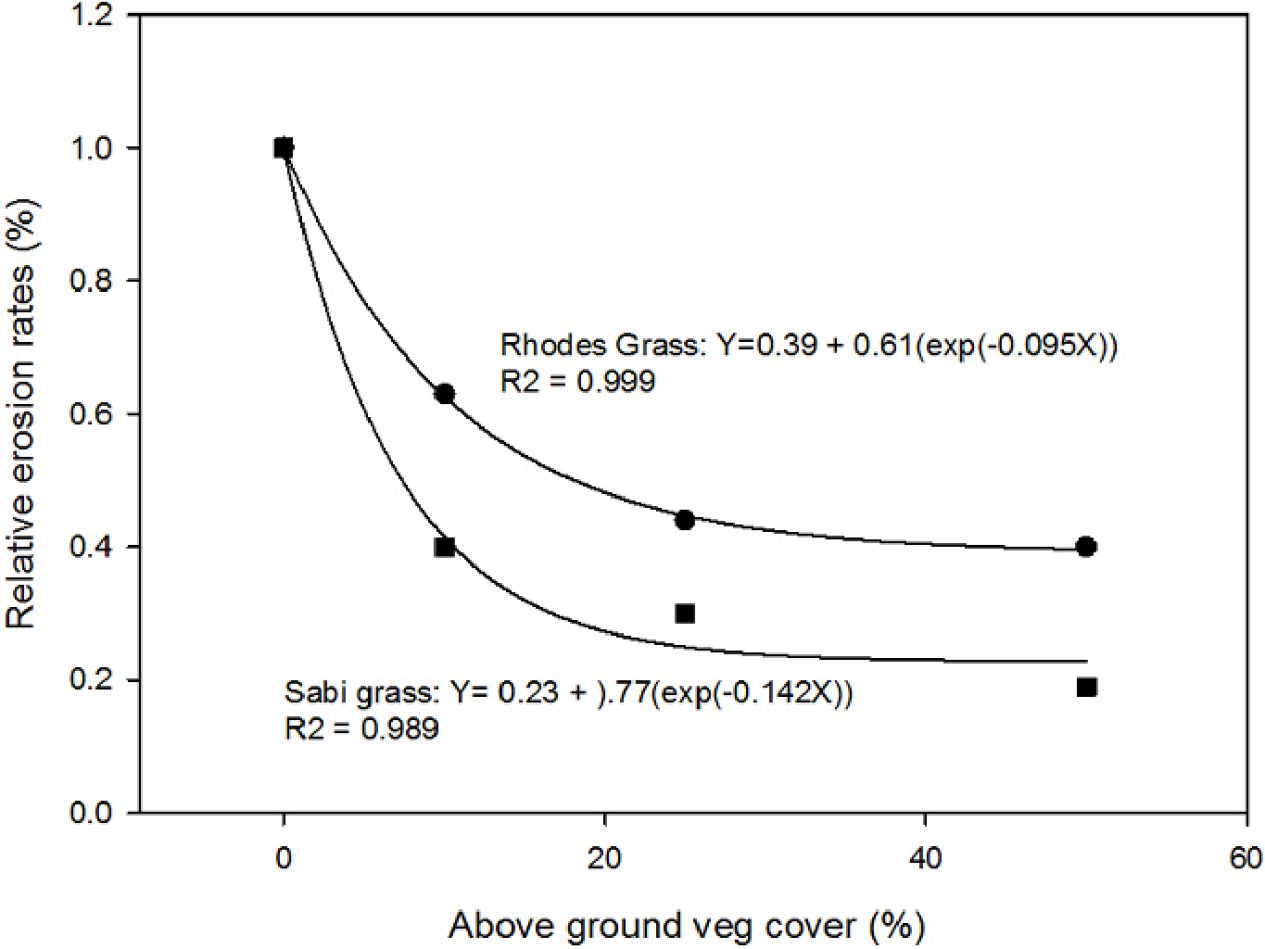
Relative soil loss as affected by vegetation type (tussocky Rhodes vs stoloniferous Sabi grasses) [13].

The Analysis of Variance (AOV) shows there were no differences in the steady state (SS) run-off (RO) rates between the combinations of soil, slope and vegetative cover, but the SS sediment concentration and hence SS erosion rate increase as veg cover decrease and slope increase. However, when erosion rate is expressed as a ratio in comparison with that from bare soil there were no differences due to soil and slope and the ratios were averaged across different soils and slopes. There was, however, a significant effect of vegetation type (see S1 File). These ratios represent the reduction in erosion rate due to vegetation cover. These ratios were plotted against the vegetation cover in percent in Fig 6.

The data in Fig 6 are independent of consolidation because the plants were grown in the insert for the same period (six months) before the plants were thinned to the required degree of vegetation cover. This graph shows that 40% of vegetation cover is required to provide the maximum erosion reduction irrespective of the type of grass established. The data also show clearly that stoloniferous grasses that creep over the ground surface such as sabi grass offers greater protection and reduction of erosion compared to tussocky grasses such as Rhodes grass.

#### Consolidation factor C_cons_

**Fig 7:**
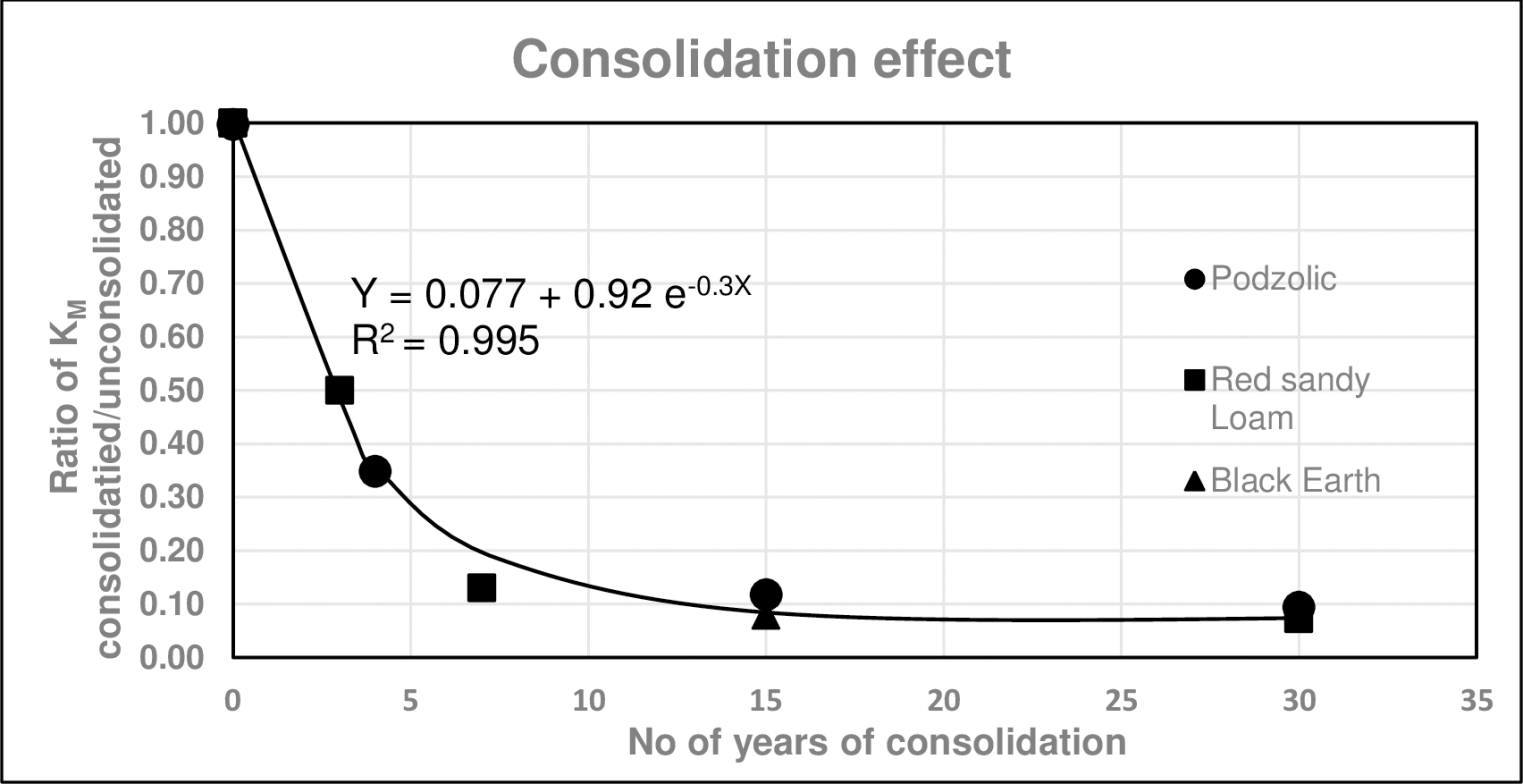
Effect of consolidation on decreasing soil erodibility (K_MUSLE_). Consolidation were a result of repeated wetting and drying and the presence of roots but not the above ground vegetation. (adapted from [17, 18]).

The relative K_MUSLE_ (K_consolidated_/K_unconsolidated_) is plotted against time (years) of consolidation in Fig 7. Measurements were taken after all above-ground vegetation cover were removed. Data were derived for a Podzolic sandy loam, rocky red sandy loam and a heavy black earth soil with clay contents ranging from 14 to 57%. These data points can be described by a single relationship:

$$Y=0.078+0.92e^{-0.3X},\;R^{2}=0.995$$ (4)

where Y is the ratio of K_consolidated_/K_unconsolidated_ and X time (years) since rehabilitation. Hence it is reasonable to assume that this relationship should also apply to most other soils as well. Fig 7 shows that it takes about 10 to 15 years for rehabilitated landscapes to reach the maximum consolidation and for erosion to be reduced by 90%. Both Figs 6 and 7 demonstrate that landscapes are most vulnerable to erosion damage during the first few years following rehabilitation and prior to the ecosystem achieving stability and maturity.

### Annual soil loss validation against field experiments

At the Curragh and Oakey Creek mines, 6 years of data were available to estimate the average annual soil loss from 20 m long bare plots. At Curragh the first 3 years were below the average rainfall followed by 3 years of above average rainfall. The long term mean EI30 was used for MINErosion 3 prediction.

At Kidston Goldmine, there was only one year of complete set of data available from the 3^rd^ year of the field trial. They were derived from 120 m bare plots(20^o^ slope); 3 years rehabilitated and vegetated plots on 20^o^ (120 m long) and 37^o^ (70 m long) slopes, and 9 years of rehabilitated and vegetated plots on 17^o^ and 37^o^ slopes. With one year of data, the measured EI30 for that year was used which amounted to 79% of the mean annual EI30 (calculated from long term data)

**Fig 8:**
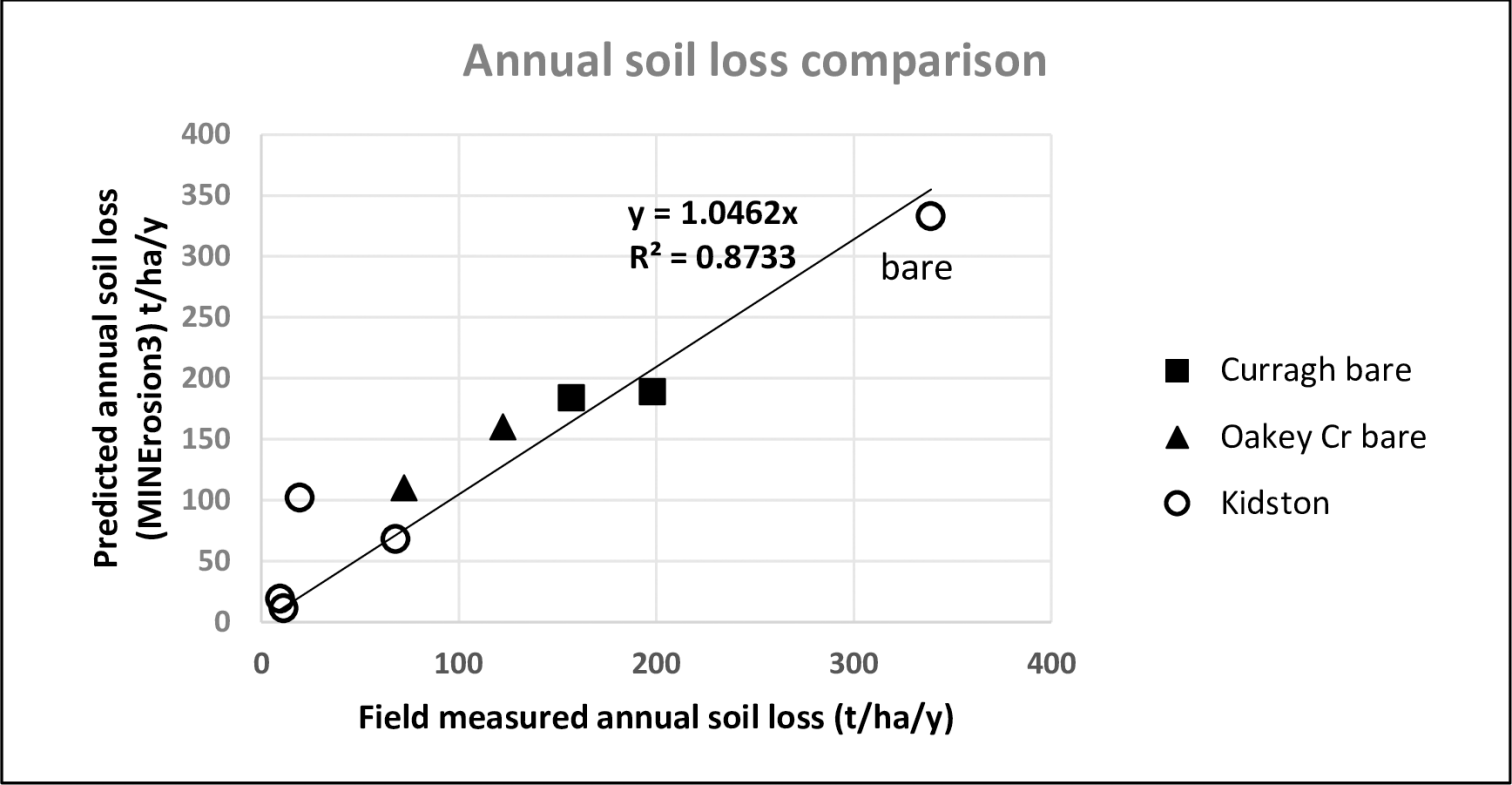
Comparison of predicted and measured annual soil loss using MINErosion 3 from three field trials in Central Queensland. Data for Curragh is an average of 6 years and Oakey Creek is an average of 4 years [3] and the data for Kidston is for one year only (bare soil and vegetated plots of 3 and 9 years).

The comparison is plotted in Fig 8 where the linear regression (Y = 1.0519X) is very close to the 1:1 line with an R^2^ of 0.86** and the Nash–Sutcliffe model efficiency coefficient value is 0.755 indicating good agreement between predicted and measured values of the annual soil loss. Although data were limited and of a short term nature, it covered a wide range of conditions and this shows that MINErosion 3 can be used to predict the annual soil loss from field plots with reasonable confidence using data derived from laboratory flumes under simulated rainfall.

#### Event based erosion validation against field experiments at Curragh

As MINErosion 3 assumes a steady state condition with a constant 100 mm.hr^-1^ rainfall intensity, the observed storm events were replaced with equivalent 100 mmhr^-1^ rainstorms with a duration that would result in the same EI_30_ as the observed storm events. Only uninterrupted single peak rainstorms with peak intensities greater than 60 mm.hr^-1^ were selected for validation of the model. Eight major single peak storm events for Curragh from 1996 to 1999 were considered suitable for validation. Storms with multiple peaks were not included at this stage. The rainfall data collected by the data logger on the Curragh mine plot areas include the total amount of rainfall, date, duration, the total energy and EI_30_ values.

The occurrence of the 8 storms on the various treatments (bare and vegetated plots) resulted in thirteen (13) erosion events from plots covered with soil (Table 1) and twenty-five (25) events from plots covered by spoil (Table 2) where complete data were available for validation. The model was run for each plot and the predicted values were plotted against the measured erosion values. A simple regression analysis was used to determine the relationship between predicted and measured values.

**Table 1:** The selected storm events for soil’s covered plots used to validate MINErosion 3.1. [25] Treatments were SoBa: Bare plots covered with soil; SoTr: Plots covered with soil and with trees as a vegetation cover; SoPa: Plots covered with soil and with pastures as a vegetation cover, S %: slope %.

Storm event date	Date of ending	Start time	Finish time	S (%)	Treatment	EI_30_	Veg Cover C (%)	Measured erosion (t.ha^-1^)	Predicted erosion (+ veg cov % & Consolidation) (t.ha^-1^)
8/1/96	10/1/96	20.04.00	6.16.00	10	SoTr	69.8	55.73	6.65	4.646
8/1/96	9/1/96	5.05.00	12.48.00	20	SoPa	69.8	44.53	10.77	8.881
8/1/96	9/1/96	5.05.00	12.48.00	20	SoTr	69.8	36.8	24.4	10.15
8/1/96	9/1/96	5.05.00	12.48.00	20	SoBa	69.8	M	26.28	42.29
8/1/96	10/1/96	6.23.00	2.32.00	30	SoPa	69.8	47.73	24.13	15.05
8/1/96	19/1/96	6.23.00	2.32.00	30	SoTr	69.8	36.53	25.49	17.2
12/12/96	12/12/96	6.37.00	12.00.00	20	SoBa	29.8	6.2	27.8	36.46
12/12/96	12/12/96	6.50.00	11.45.36	30	SoTr	29.8	65.3	14.1	12.54
29/12/98	30/12/98	18.30.00	6.46.00	10	SoTr	334.7	100	0.1	9.364
29/12/98	30/12/98	18.20.00	3.28.00	20	SoBa	334.7	0	73.9	61.92
29/12/98	30/12/98	18.42.00	9.46.00	30	SoPa	334.7	100	M	13.09
29/12/98	30/12/98	18.42.00	9.46.00	30	SoTr	334.7	99	M	13.09
10/6/99	10/6/99	0.08.00	11.44.00	20	SoBa	40	0	25.1	17.26

**Table 2:** The selected storm events for spoil’s covered plots used to validate MINErosion 3.1. [25]. Treatments were SpBa: Bare plots covered with spoil; SpTr: Plots covered with spoil and with trees as a vegetation cover; SpPa: Plots covered with spoil and with pastures as a vegetation cover, S %: Slope %.

Storm event date	Date of ending	Start time	Finish time	S (%)	Treatment	EI_30_	Veg Cover C (%)	Measured erosion (t.ha^-1^)	Predicted erosion (+ veg cov % & Consolidation) (t.ha^-1^)
8/1/96	9/1/96	5.05.00	12.48.00	20	SpBa	69.8	M	80.44	71.008
8/1/96	9/1/96	5.05.00	12.48.00	20	SpTr	69.8	3.73	45.97	71.008
8/1/96	9/1/96	5.05.00	12.48.00	20	SpPa	69.8	0.27	50.7	71.008
8/1/96	10/1/96	6.23.00	2.32.00	30	SpTr	69.8	10.4	60.42	52.063
30/4/96	1/5/96	4.46.00	11.00.00	20	SpBa	10.3	7.8	16.7	36.289
30/4/96	1/5/96	4.46.00	11.00.00	20	SpTr	10.3	7.6	14.8	36.289
30/4/96	1/5/96	4.46.00	11.00.00	20	SpPa	10.3	7.5	15.7	36.289
8/10/96	8/10/96	15.54.00	20.48.00	10	SpTr	6.3	13.4	9.6	4.174
8/10/96	8/10/96	15.54.00	20.48.00	10	SpPa	6.3	45.1	2.2	2.191
8/10/96	9/10/96	15.16.00	0.58.00	20	SpBa	6.3	0.2	10.3	29.309
8/10/96	9/10/96	15.16.00	0.58.00	20	SpTr	6.3	4.4	24	29.309
8/10/96	9/10/96	15.16.00	0.58.00	20	SpPa	6.3	0.9	18.7	29.309
12/12/96	12/12/96	6.48.00	12.15.00	10	SpTr	29.8	5.5	14.8	15.291
12/12/96	12/12/96	6.48.00	12.15.00	10	SpBa	29.8	30.3	8.2	3.67
12/12/96	12/12/96	6.37.00	12.00.00	20	SpBa	29.8	0	28.3	36.671
12/12/96	12/12/96	6.37.00	12.00.00	20	SpTr	29.8	4.1	23.7	36.671
12/12/96	12/12/96	6.37.00	12.00.00	20	SpPa	29.8	0.3	27.1	36.671
22/3/97	24/3/97	18.37.00	9.45.00	20	SpBa	*	0	57.1	18.671
22/3/97	24/3/97	19.19.00	10.24.00	30	SpTr	*	1	48.8	37.894
22/3/97	24/3/97	19.19.00	10.24.00	30	SpPa	*	1	52.2	37.894
29/12/98	30/12/98	18.30.00	6.46.00	10	SpTr	334.7	5	26	40.042
29/12/98	30/12/98	18.30.00	6.46.00	10	SpPa	334.7	30	8.3	9.61
29/12/98	30/12/98	18.20.00	3.28.00	20	SpBa	334.7	0	56.7	50.119
10/6/99	10/6/99	1.05.00	7.41.00	10	SpTr	40	5	10.3	9.119
10/6/99	10/6/99	1.05.00	7.41.00	10	SpPa	40	20	3.5	3.647

**Fig 9:**
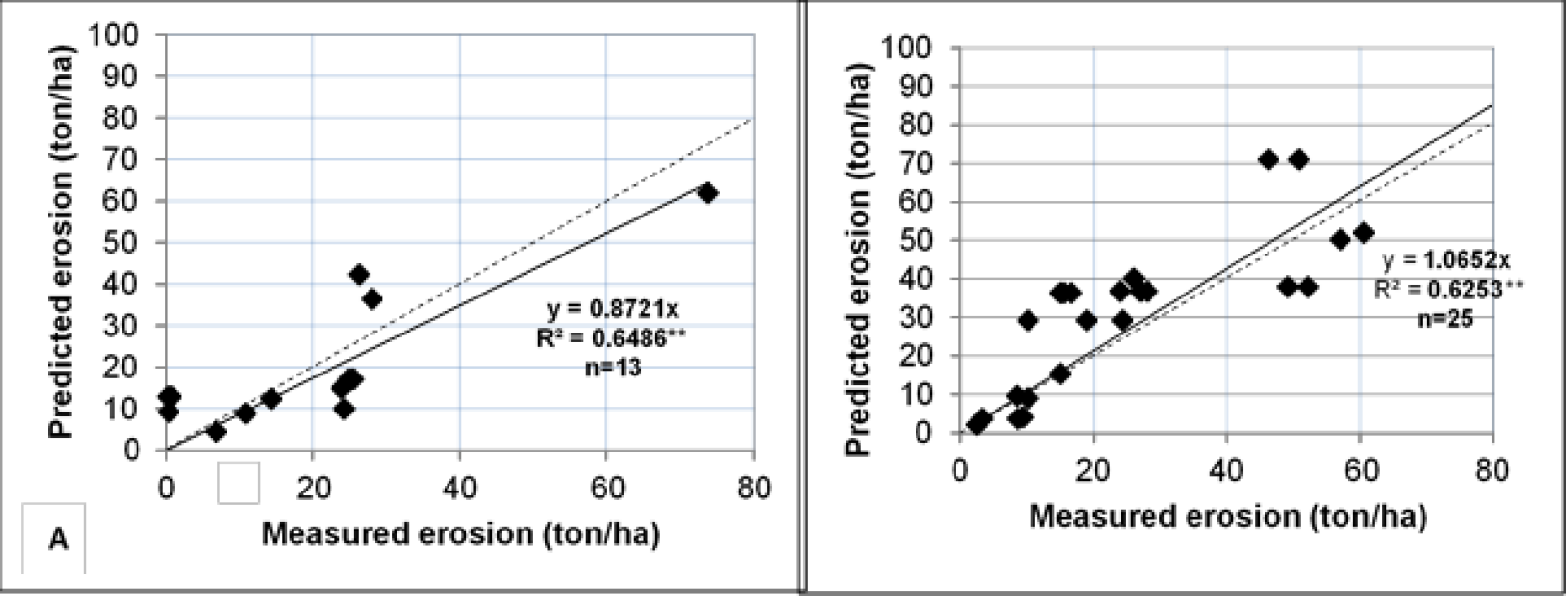
**Predicted (using MINErosion 3.4) and measured erosion rate values for soil (A) and overburden spoils (B) plots** [25]. Dotted lines are the 1:1 lines.

Fig 9 shows the regression between measured (X) and predicted (Y) values for all soil plots (A) with a linear equation through the origin (Y = 0.872 X) and an R^2^ of 0.649**. A Nash and Suttcliffe coefficient of efficiency [26] was calculated as 0.865 While a similar comparison for all overburden spoil plots (Fig 9B) shows the regression as Y = 1.065 X with an R^2^ of 0.625** and a Nash Sutcliffe coefficient of efficiency of 0.612 [25]. Considering the simplicity of the model and the few parameters required to drive the model, the prediction accuracy is reasonably good. On average, MINErosion 3 predicted 87.2% of the measured erosion on the soil plots and 106.5% on the overburden plots.

## Discussion

MINErosion 3 was developed as a model to predict potential event based and annual erosion rates from steep rehabilitated post-mining landscapes under intense tropical rainstorms, based on measurements conducted in the laboratory under simulated rainfall. As laboratory measurements can be conducted prior to landscape reconstruction or even prior to commencement of mining, it provides mine-sites with the ability to estimate the rate of annual and event based erosion from any proposed post-mining landscapes, thus providing various options for post mining rehabilitation planning. Results from this work shows that the prediction of erosion rates from defined post-mining landscapes are accurate and reliable.

The assumptions that underpin MINErosion 2 and 3 are that run-off and soil loss processes following intense tropical rainstorms on uniform, unconsolidated, bare soil/spoil on slopes of low surface roughness, can be modelled as a time-invariant steady-state process [1]. Under the intense tropical rainstorms, these assumptions are reasonable as steady state conditions will be quickly established. Only rill and inter-rill processes are considered while the effect of tunneling and gullying are not included. Rills are assumed to flow perpendicular to the contour with equal flow in each rill which are assumed to occur at a density of 1 rill.m^-1^ based on analysis of rill density data by Gilley et al. [27] and our observations of average rill densities in the field are consistent with that. The separation of rill and inter-rill erosion overcomes the problem of scaling from small to large plots. As inter-rill sediment feeds into the rills, they are insensitive to slope length. However, as slope length increases, rill erosion increases significantly. Therefore, the contribution of rill and inter-rill sediment to total soil loss varies with slope length. Errors may be introduced if sediment deposits within the rill which may happen if rainfall intensity is low or slopes too long. However under high intensity tropical rainstorms all sediments are likely to be transported to the bottom of the rill.

As raindrop kinetic energy is a major driving force for erosion, in the validation of the model for event-based erosion, the predicted erosion rate was calculated for a design storm of 100 mm.hr^-1^ with a duration that would result in the same EI30 value as that of the observed storm. Run-off properties were estimated from this design storm and the soils infiltration rates, while the vegetation cover used was measured on the field plots and consolidation estimated from the age of rehabilitation. Similarly for the annual soil loss, the mean cumulative annual EI30 was used. The agreement between predicted and measured event and annual erosion rates were excellent, confirming that the assumptions used in this model are reasonable, in particular that erosion from intense tropical rainstorms can be modelled as steady state processes.

**Fig 10:**
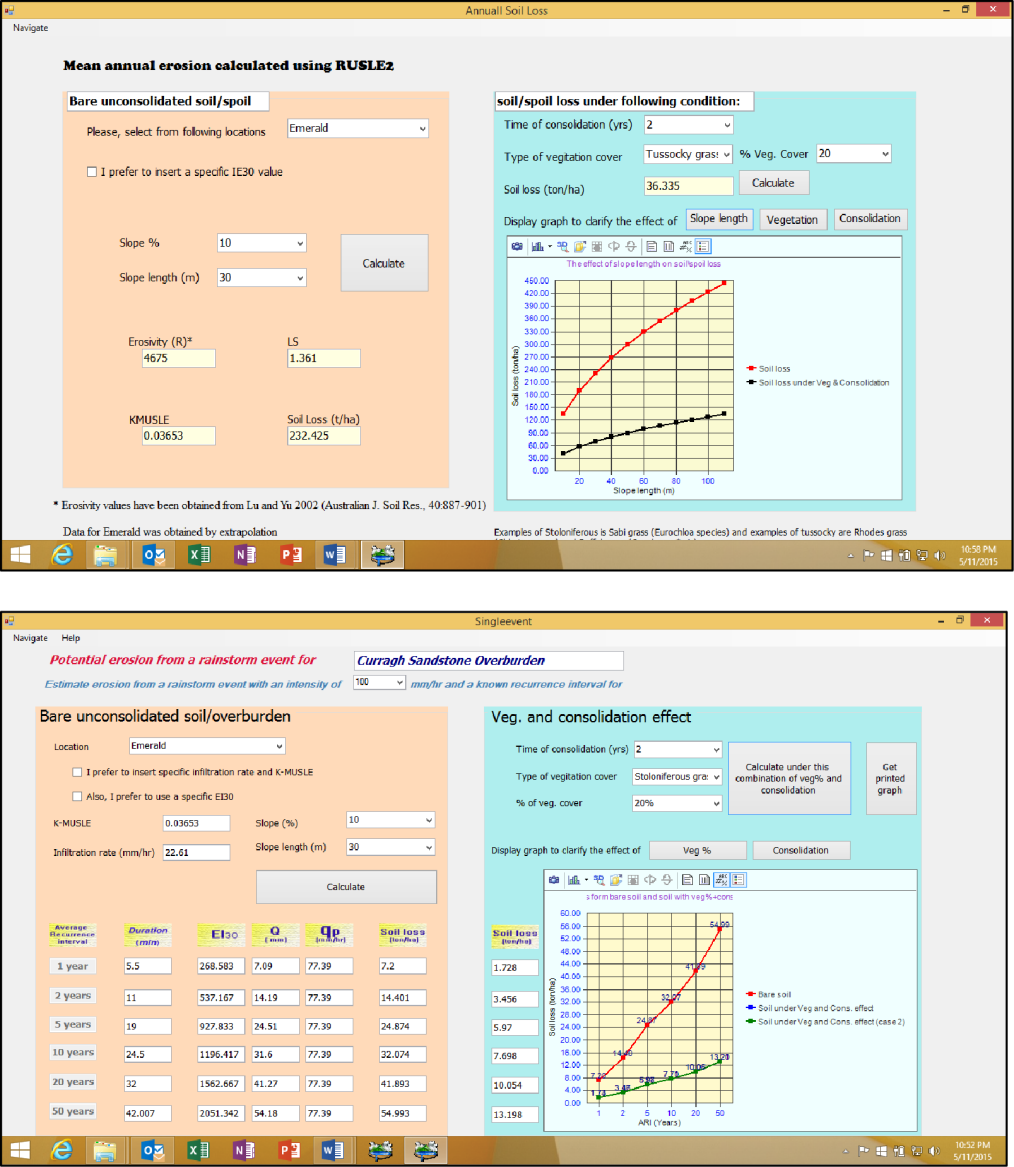
**Example of annual (top) and event soil loss (bottom) output pages**.

As the main target users of this model are busy environmental officers with limited available time, MINErosion 3 was designed as a user friendly model to derive appropriate landscape design parameters. Detailed data of 35 soil and spoil materials from Central Queensland mines are embedded in the model. They can be readily viewed and are available to the user to select from. For soil or spoil from areas other than Central Queensland, the user can select a soil/spoil from the database with properties that is closest to the soil/spoil of interest. Alternatively they can use their soil/spoil physical and chemical properties to estimate the interrill and rill erodibilities, or they can make their own measurements (detailed procedures embedded in the model) and processed the data through the MINErosion 3 model. Detailed explanatory notes were embedded in the model to enable users to familiarize themselves quickly or to refresh on the details of the model. This includes an introduction to erosion and erosion modelling, procedures used for conducting laboratory and field rainfall simulation, and procedures for soil physical and chemical analysis used to derive the parameters used in the model. Other target users are students and the embedded features of the model would also be useful as an educational tool. Outputs as graphs and/or tables can readily be obtained and printed. Fig 10 shows examples of outputs for annual and event soil loss.

Fig 10 shows a sample of the default calculation of soil loss from bare unconsolidated soil or spoil on the left side of each page, and soil loss estimation from consolidated and vegetated soil or spoil are displayed on the right side of each page. In each case, graphical outputs are limited to two, i.e. one on the bare unconsolidated plot (default case) and one the other case. Despite these limitations, the model is readily repeated for different scenarios and the results plotted to suit the need at the time.

The question of acceptable erosion rates is probably best defined as the average erosion rates of the surrounding natural unmined areas. These can be estimated by using MINErosion 3 with consolidation factor of 10 years or more (Fig 7) with an appropriate vegetation cover representing the natural unmined surrounding areas. In the case of Curragh soil in Fig 10, if undisturbed with a 10% slope gradient, 30 m slope length and 50% vegetation cover, the annual average erosion rates would be approximately 10.5 t.ha^-1^.year^-1^.

Table 3 shows an example of estimated annual erosion rate scenarios from a range of possible rehabilitation scenarios with the Curragh soil. It is clear that erosion rates from a 10% slope remained too high and will take approximately 10 years with at least 20% vegetation cover to reduce to acceptable levels. However, if slopes are reduced to 6%, erosion rates are reduced to acceptable levels if vegetation cover of 20% can be achieved with stoloniferous grasses. Additional scenarios can be generated to enable appropriate decisions to be made on the optimum slope gradient and slope length for acceptable erosion control. A similar set of scenarios can be readily generated for event erosion rates to assess the risk of potential erosion damage to rehabilitated landscapes.

**Table 3:** Predicted annual erosion rates for a rehabilitated and vegetated postmining hillslope covered with Curragh soil. Vegetation options are tussocky or stoloniferous grasses. Shaded cells have less than 12.5 t/ha/y.

**slope**	**10% slope**								
	**0 year**	**2 years consolidation**				**10 years consolidation**			
	**bare**	**Tussocky grass cover**		**Stoloniferous grass cover**		**Tussocky grass cover**		**Stoloniferous grass cover**	
**Veg cover**		**20%**	**40%**	**20%**	**40%**	**20%**	**40%**	**20%**	**40%**
** Length(m)**									
10m	131	39	31	19	16	**7**	**5**	**3**	**3**
20m	185	55	44	27	22	**9**	**7**	**4**	**4**
30m	226	68	54	33	27	**11**	**9**	**5**	**5**
40m	261	70	63	38	31	13	**10**	**6**	**5**
50m	292	88	70	42	35	15	**12**	**7**	**6**
									
** slope**	**6% slope**								
** **	**0 year**	**2 years consolidation**				**10 years consolidation**			
	**bare**	**Tussocky grass cover**		**Stoloniferous grass cover**		**Tussocky grass cover**		**Stoloniferous grass cover**	
**Veg cover**		**20%**	**40%**	**20%**	**40%**	**20%**	**40%**	**20%**	**40%**
**Length (m)**									
10m	64	19	15	**9**	**8**	**3**	**3**	**2**	**1**
20m	90	27	22	13	**11**	**5**	**4**	**2**	**2**
30m	111	33	27	16	13	**6**	**4**	**3**	**2**
40m	128	38	31	18	15	**6**	**5**	**3**	**3**
50m	143	43	34	21	17	**7**	**6**	**3**	**3**

Another possible use of MINErosion 3 is to estimate erosion onto the mine working area. On one side of the working pit of an open-cut coal mine is the newly formed overburden spoil pile at the angle of repose (75%) and could be well over 50 m high, and is therefore vulnerable to erosion from intense rainstorms. This could affect the working pit conditions. The probability of these severe erosion event from the recurring rainstorms is shown in Table 4 for Curragh sandstone overburdens of 60 m slope lengths at 60% slope. The data shows the risk of severe erosion events from the steep slopes of spoil piles and how vegetation and consolidation can significantly reduce erosion, but they remain very high and require other measures to control erosion and prevent damage.

**Table 4:** Storm event erosion rates (t/ha) from Curragh sandstone overburdens at a slope of 60% and a slope length of 60 m under different consolidation and vegetation cover. The average recurrence intervals used was for the Emerald region of central Queensland.

Average storm Recurrence interval (1:x years)	Duration of 100 mm/h storm (mins)	Erosion rates (t/ha/event)		
		Unconsolidated	2 years consolidation	
		Bare	Bare	30% veg cover
**1**	**5.5**	**1615**	**969**	**232**
**2**	**11**	**3230**	**1938**	**465**
**5**	**19**	**5580**	**3348**	**803**
**10**	**24.5**	**7195**	**4317**	**1036**
**20**	**32**	**9398**	**5638**	**1353**
**50**	**42**	**12337**	**7402**	**1776**

A unique feature of this model is an embedded database of soil physical and chemical properties and erodibilities of 35 materials i.e. 17 soils and 17 overburdens from open-cut coalmines and 1 soil from an open-cut goldmine in Central Queensland (CQ), which can readily be selected from a pull-down menu. This covers a wide range of soils and spoils from Central Queensland (CQ) and enables environmental officers to readily make erosion predictions for most CQ mine-sites. For Australian soils or overburdens that are not covered by this database, additional measurements can be made with a tilting flume-rainfall simulator facility similar to that available at Griffith University, to determine the rill and inter-rill erodibilities of the new material, and they can be entered into the database. Alternatively, they can be estimated (as a first approximation) from routine soil physical and chemical properties (Sheridan et al., 2000a) and these options are built into the model. As the climatic data in the current model is limited to the Australian continent, for locations outside the Australian continent, the annual EI30 can be used to estimate the average annual soil loss, but local climatic data (frequency intensity duration of rainstorms) is required to determine storm event erosion with specific recurrence intervals.

The current version is the MINErosion 3.4 and a copy of the installation file is available for download from the authors (h.so@griffith.edu.au; ashraf.khalifa@gmail.com) or downloaded from the following link: https://www.dropbox.com/s/oq4487w7btd7r1u/setup.exe?dl=0.

The model has not been adequately tested for use on very low slopes and cropping land (< 6%). Further work will be required to check the validity of the model for these conditions. As MINErosion 3.4 is a hillslope erosion model, it cannot be used to estimate the total erosion from a catchment or the overall post-mining landscape with multiple catchments. For that, MINErosion 3.4 needs to be combined with a GIS system (ArcGIS) into a landscape erosion model (MINErosion 4) and this will be presented and discussed in a following paper.

The model’s vegetation cover is limited to grass vegetation cover at this stage. No data are available for shrubs or tree cover and further measurements and work is required to develop appropriate cover functions for such vegetation.

## Conclusions

In conclusion, this paper has shown that the hillslope erosion model MINErosion 3.4 can be used to estimate the expected mean annual erosion rate and event erosion rates from steep post-mining landscapes using parameters measured on laboratory tilting flumes under simulated rainfall. As MINErosion 3.4 is based on steady state processes, erosion on steep landscapes under intense tropical rainstorms are adequately approximated and modelled by steady state processes. Finally, MINErosion 3.4 is a user friendly application that allows mine-sites to readily derive key landscape design parameters (slope gradient, slope length and vegetation cover) to construct post-mining landscapes that will result in acceptable erosion rates. MINErosion 3.4 prediction was validated against measured annual erosion rates and event erosion rates from field erosion plots on rehabilitated post-mining landscapes at two open-cut coalmines and one open-cut goldmine in Central Queensland with excellent agreement. The software MINErosion 3.4 allows mine-sites to estimate mean annual erosion rates from proposed or constructed post-mining landscapes, as well as erosion rates from rainstorms with selected average recurrence intervals and use this as input into their risk analysis for potential erosion damage assessment.

## Supporting information

S1 File Determination of the above ground vegetative cover factor. (DOCX)

S2 File Determination of the consolidation factor. (DOCX)

S3 File Procedures used for validation of annual and individual rainstorm erosion events. (DOCX)

## References

[1] Sheridan, G. J. Predicting Hillslope Scale Erodibility and Erosion on Disturbed Landscapes from Laboratory Scale Measurements. PhD thesis 2001, University of Queensland, St Lucia, Queensland, Australia

[2] Williams D.J. Management of solid wastes in Mulligan D.R.(Ed) Environmental Management in the Australian Minerals and Energy Indusries. Principles and Practices. UNSW Press and AMEEF 1996, pp 157–188.

[3] Carroll, C., Pink, L. & Burger, P. Coalmine Rehabilitation: A long term erosion and water quality study on Central Queensland coalmines ISCO 2004–13th International Soil Conservation Organization Conference, Conserving Soil and Water for Society: Sharing Solutions. July 2004, July 2004. 2004 Brisbane.

[4] Domagala, J. & Wilson, I. Land: Mining disturbance. In: Freeman, J. & Webber, W. (eds.) State of the Environment Queensland 2007. Brisbane.

[5] DEHP. Guideline Financial assurance under the Environmental Protection Act 1994 ESR/2015/1758• Version 3.00, Effective: 4 MAR 2016 State of Queensland https://www.ehp.qld.gov.au/assets/documents/regulation/era-gl-financial-assurance-ep-act.pdf

[6] Wischmeier W. H. & Smith D. D. 1960 A universal soil-loss equation to guide conservation farm planning. Trans.7th Int. Congr. Soil Sci., 1960, 418–425.

[7] Renard K.G., Foster G.R., Weesies G.A., Porter J.P. Revised Universal Soil Loss Equation. J. Soil Water Cons. 1991: 46, 30–3

[8] Flanagan D. C. & Laflen J. M. The USDA Water Erosion Prediction Project (WEPP). Eurasian Soil Science, 1997: 30, 524–530

[9] So, H.B.; Sheridan, G.J., Horn, C.P. and Currey, N.A. Predicting hillslope scale erosion on, landscapes from laboratory scale measurements. In Mohtar, RH (Ed) The global farm: Selected papers from the 1999 ISCO meeting 2001, p 1053–1058.

[10] Sheridan G. J., So H. B., Loch R. J. & Walker C. M. Estimation of erosion model erodibility parameters from media properties. Special Issue: Aust. J. Soil Res. 2000a: 38, 265–284.

[11] Sheridan G. J., So H. B., Loch R. J., Pocknee C. & Walker C. M. Use of laboratory-scale rill and interill erodibility measurements for the prediction of hillslope-scale erosion on rehabilitated coal mine soils and overburdens Special Issue: Aust. J. Soil Res.,2000b: 38, 285–97.

[12] Onstad C.A. Foster G.R. Erosion modeling on a watershed. Trans. Am. Soc. Civ. Eng. 1975: 26, 1102–4, 1108.

[13] Ferguson, K. Using rainfall simulation to assess the effects of consolidation and ground cover on rates of soil loss. Honors thesis 2002, BLandRSc degree, University of Queensland, St Lucia, Queensland, Australia

[14] Wischmeier, W.H. & Smith, D.D. Predicting rainfall erosion losses. A guide to conservation planning. Agricultural Handbook no. 537.USDA-SEA 1978, US. Govt. Printing Office, Washington, DC.

[15] Foster, GR; Toy, TE & Renard, KG (2003). Comparison of the USLE, RUSLE1.06c and RUSLE2 for application to highly disturbed lands. 2003. Available at http://www.tucson.ars.gov//icrw/proceedings/foster.pdf

[16] Loch R. J., Robotham B. G., Zellar L., Mastermann N., Orange D. N., Bridge B. et al A multi-purpose rainfall simulator for field infiltration and erosion studies. Australian Journal of Soil Research 2001: 39: 599–610.

[17] Horn, C.P., Yatapanage, K., So, H.B., Mulligan, D.R. Erosion from rehabilitated and unmined sites at Blair Athol Mine. Final Report to Blair Athol Mine 2001, Center for Mined Land Rehabilitation, The University of Queensland, St Lucia, Queensland, Australia

[18] Horn, C.P., Kopittke, G.R., Williams, S., Yatapanage, K., So, H.B., Mulligan, D.R. Erosion from rehabilitated and unmined sites at Mount Isa Mines. Final report to MIM Holdings 1999, Center for Mined Land Rehabilitation, The University of Queensland.

[19] So, H.B., Yatapanage, K.G. (2004) MINErosion 3.01: A user friendly integrated package for monitoring and prediction of potential rates of soil erosion from steep mine sites. In Eds. SR Raine, AW Biggs and NW Menzies, DW Freebairn and P Tolmie. Conserving Soil and Water for Society. Sharing solutions. Proc. 13th International Soil Conservation Organisation Conference 2004 4-9th July, Brisbane. ASSSI/IECA. Paper 403. pp 5.

[20] Carroll, C. Temporal Changes In Runoff and Erosion Processes on Disturbed Landscape Under Natural Rainfall. PhD thesis 2005, University of Queensland, St Lucia, Queensland, Australia.

[21] Canterford R. P., Pescod N. R., Pearce H. J. & Turner L. H. Design Intensity—Frequency—Duration Rainfall In: PILGRIM D. H. (ed.) Australian Rainfall and Runoff: A Guide to Flood Estimation 1987 Barton, ACT: Institution of Engineers, Australia.

[22] Bureau of Meteorology. Intensity Frequency Duration design Rainfall 2016. Available at: www.bom.gov.au/water/designRainfalls/ifd

[23] So, H. B., Sheridan, G. J., Loch, R. J., Carroll, C., Willgoose, G., Short, M. et al. Post-mining Landscape Parameters for Erosion and Water Quality Control. Final report 1998 on ACARP projects 1629 and 4011. The University of Queensland, St Lucia, Queensland, Australia

[24] Horn, C.P., Yatapanage, K.G., So, H.B., Mulligan, D.R. Waste Rock Dump Research Project: Stability of outer dump surfaces. Kidston Gold Mine-Dump slope erosion Final Report 2000. Center for Mined Land Rehabilitation, University of Queensland. St Lucia, Queensland, Australia

[25] Khalifa, A. M. MINErosion 4: A user-friendly catchment/landscape erosion prediction model for post mining sites in Central Queensland. PhD thesis 2010, Griffith University, Nathan, Queensland, Australia.

[26] Nash J.E., Sutcliffe J.V. River flow forecasting through conceptual models. Part 1: A discussion of principles. Journal of Hydrology 1970: 10, 282–290.

[27] Gilley J. E., Kottwitz E. R. & Simanton J. R. Hydraulic characteristics of rills. Trans. Am. Soc. Agri. Eng.,1990: 33, 1900–6

