# Supplementary material for: MINErosion 3: Using measurements on a tilting flume-rainfall simulator facility to predict erosion rates from post-mining landscapes in Central Queensland, Australia
Source: PLoS One. 2018 Mar 28;13(3):e0194230. doi: 10.1371/journal.pone.0194230 (PMC5874007; doi:10.1371/journal.pone.0194230)
Supplement: S1 File — (DOCX) [file pone.0194230.s001.docx]

**Supporting documents:**

**S1: Determination of the above ground vegetative cover factor.**

**Objective:** The objective of this study was to determine the effects of above ground vegetative cover on the erodibilities of spoil and soil.

**Facilities**: The study was conducted on the tilting flume-rainfall simulator facility in the Erosion Processes Laboratory at the University of Queensland (Fig 1). This facility had several inserts (3m long x 0.8 m wide x 0.25m deep) that can be lifted into and out of the flume allowing simulation to be repeated after treatments are applied to the flume inserts. Some inserts are split in the centre as shown on the left picture of Fig 1. Grass can be gown to simulate different vegetative cover (Fig 2). As vegetation grows and the soil/spoil goes through multiple wetting and drying cycles, it will be subjected to a process of consolidation (an increase in bulk density and strength). The above ground vegetation can be trimmed to simulate different degrees of cover without affecting the soils erodibility.


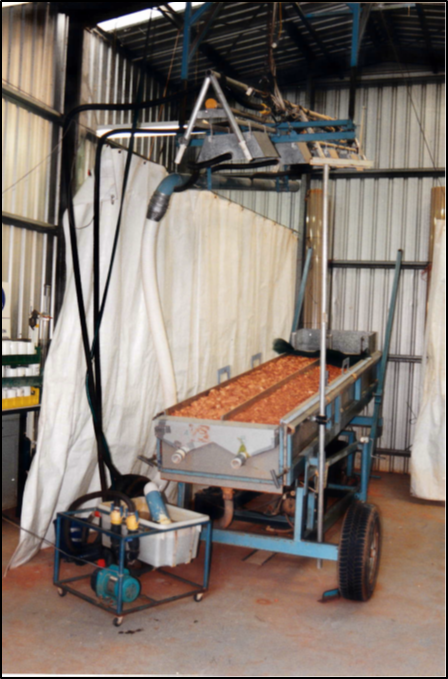

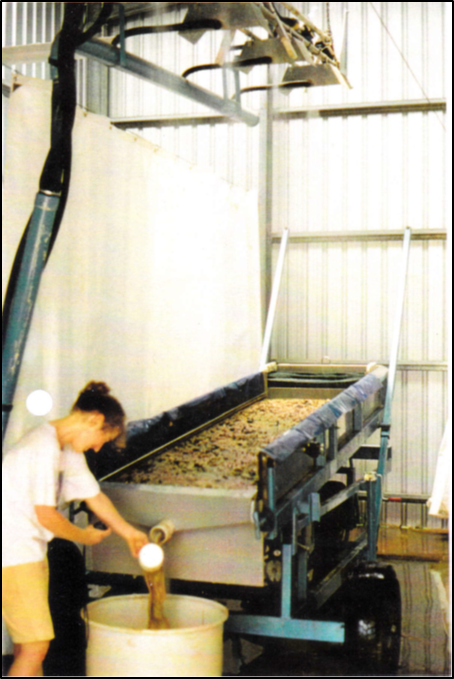


**Fig 1 : The tilting flume and oscillating rainfall simulator facility. On the left the flume was split in the center and fitted with a twin-collector. On the right it shows the manual collection of run-off and sediment to determine their rates. No inserts were used in this picture.**


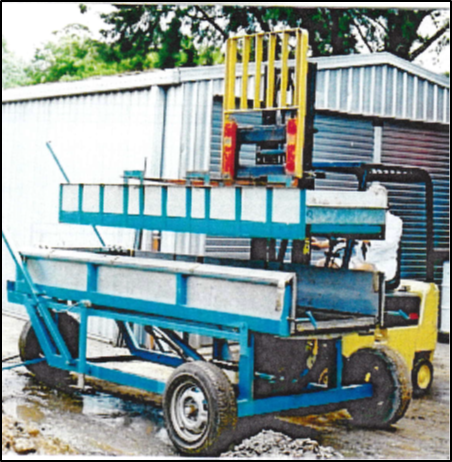
 0
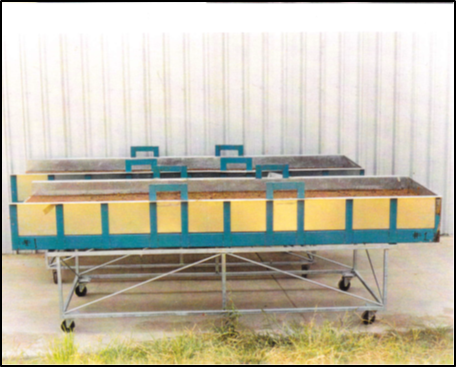


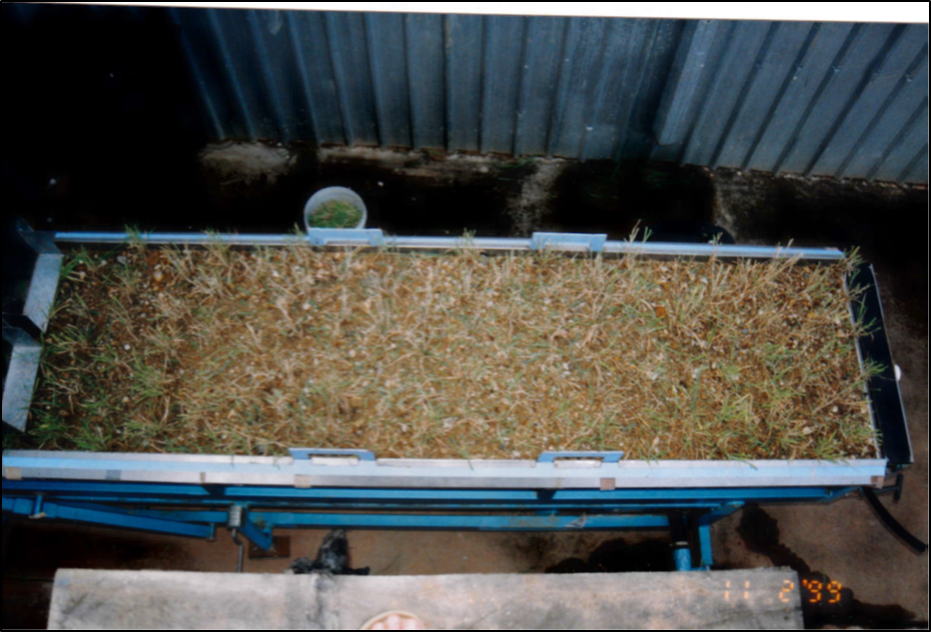


**Fig 2: The inserts for the flume. The top left picture shows the mobile flume with an insert ready to mount inside the flume. On the top right picture are 2 flumes on mobile trolleys. At the bottom is a top view of an insert with grass growing on spoil (overburden) material.**

**Sample preparation:** To determine the effect of vegetation cover on erosion rates, 8 inserts were packed with soil to a depth of 0.15 m. 2 soils were used for 4 inserts each. The first soil was the surface soil of a Vertisol (Black Earth from the Gatton experiment station of the University of Queensland ) with 81 % clay, 11 % silt and 8 % sand, and the second soil was a grey podzolic(Ultisol) surface soil from the Mt Cotton University farm with 21 % clay, 29 %silt and 50 % sand. The inserts were sown with grass seeds and after establishment the plants were thinned to a uniform population with distances of 0.15 m between plants. Four inserts of each soil was sown with Rhodes grass (*Chloris guyana* with a tussocky growth habit) and another four with Sabi grass (*Urochloa mosambicensis* with a stoloniferous or creeping growth habit). Sabi grass is expected to be more effective in reducing erosion due to its creeping growth habit. The experimental set-up consisted of 2 soils x 2 grasses x 2 replicates for the 8 inserts. They were watered regularly, fertilised and kept weed free for 6 months to ensure good grass cover before being used for further measurements under the rainfall simulator.

**Rainfall simulation:** Rainfall simulation were performed following the procedure of Sheridan (2000) on the unconsolidated soils prior to sowing, to measure their initial erodibility before treatments were imposed. As each flume reached adequate vegetation cover, they were trimmed to a vegetation cover of 50 % for the first simulation, then to 25 %, 10 % and 0 % for the following simulations. The 0 % cover was used to determine the erodibility from the consolidated soil. Given the different growth habits of the grasses, the trimming regime for the 2 grasses have to be different and is shown in table 1. As Sabi grass expands by growing runners that ankers into the soil at several rooting points, trimming the vegetation cover is done differently from the tussocky Rhodes grass. Visual assessment using photographic standards were used to guide the process of trimming.

**Table 1: Trimming of the above ground vegetation to create different levels of vegetation cover.**

| **Veg Cover level** | **Sabi Grass** | **Rhodes Grass** |
| --- | --- | --- |
| 50 % | As is | Cut at 15 cm from base of plant |
| 25 % | Removal of 50 % of shoots after last rooting point | Cut at 10 cm from base of plant |
| 10% | Removal of another 60 % of shoots after last rooting point | Cut at 4 cm from base of plant |
| 0 % | Removal of all remaining shoots | Cut plant off at the base |

At each cover level, rainfall simulation was conducted for 30 minutes each with a rainfall of 100 mm/h, at 10, 15, 20 and 30 % slopes. Run-off samples were collected in plastic jars for 10 seconds at 3, 5, 8. 12, 16, 20 and 25 minutes and their volumes determined. Run-off rates were calculated and the suspension were then oven dried at 80^o^C to measure the sediment delivery rates or erosion rates. As steady state is reached within 15 mins, the last 3 measurements were averaged as a measure of that steady state erosion rate. The effect of vegetation cover was expressed as the ratio of steady state erosion rate relative to the erosion rate at 0% cover, the latter being equal to 1.00. At the start and end of each series of simulations, the rainfall simulator was calibrated to ensure that the rates of rainfall used remain constant.

Data were entered into an Excel based template for calculating K_Interrill_ , K_rill,_ infiltration rates and the slope adjustment factors for each soil or spoil material. These in turn are essential inputs into MINErosion 2 and 3 models.

**Results:**

There were no differences in the steady state run-off rates between the combinations of soil, slope and vegetative cover except for the heavy clay Gatton soil under 50 % veg cover where RO rates are lower. The steady state sediment concentrations increased as veg cover decreases and the slope increases. Therefore, the steady state erosion rates would also increase with a decrease in veg cover (P=0.05) and an increase in slope. The steady state erosion rates were normalised as t/ha per mm of rainfall.

The effect of vegetation cover on erosion is expressed by the ratio of erosion rates relative to 0 vegetation cover and is shown in Table 2. With no vegetation cover, the ratio is equal to 1.0. There is a tendency for Rhodes grass to show smaller reduction as vegetation cover increase. There is no significant effect of soil and slope, and therefore the data was pooled and averaged in Table 2 and plotted in Fig 4.

**Table 2: Effect of % veg cover on the ratio of erosion rates with veg/without veg.**

| **Veg** | **Rhodes grass** | | | | | | | | | **Sabi grass** | | | | | | | | |
| --- | --- | --- | --- | --- | --- | --- | --- | --- | --- | --- | --- | --- | --- | --- | --- | --- | --- | --- |
| **Cov** | **Gatton soil with slope of** | | | | **Mt Cot soil with slope of** | | | | **Ave** | **Gatton soil with slope of** | | | | **Mt Cot soil with slope of** | | | | **Ave** |
|  | **30** | **20** | **15** | **10** | **30** | **20** | **15** | **10** |  | **30** | **20** | **15** | **10** | **30** | **20** | **15** | **10** |  |
| **0** | 1.0 | 1.0 | 1.0 | 1.0 | 1.0 | 1.0 | 1.0 | 1.0 | **1.0** | 1.0 | 1.0 | 1.0 | 1.0 | 1.0 | 1.0 | 1.0 | 1.0 | **1.0** |
| **10 %** | .67 | .57 | .50 | .65 | .77 | .49 | .53 | .85 | **.63** | .40 | .45 | .28 | .30 | .56 | .40 | .40 | .50 | **.42** |
| **25 %** | .42 | .33 | .32 | .42 | .64 | .40 | .38 | .50 | **.43** | .27 | .16 | .14 | .17 | .39 | .29 | .35 | .40 | **.29** |
| **50 %** | .22 | .23 | .23 | .18 | .64 | .40 | .50 | .50 | **.36** | .18 | .11 | .07 | .09 | .17 | .14 | .25 | .30 | **.17** |


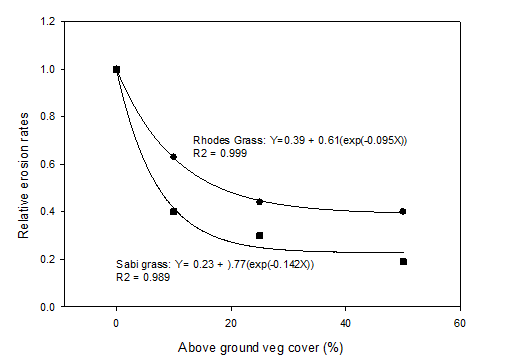


**Fig 4: The vegetative cover function expressing the effect of above ground vegetative cover (%) on the relative erosion rates (Ratio of erosion rates with/without vegetation.**

**References**:

Ferguson, K (2002): Using rainfall simulation to assess the effects of consolidation and ground cover on rates of soil loss. 4^th^ year honours thesisin Land Resources, School of Land and Food Sciences, The University of Queensland.

Sheridan, GJ; So, HB; Loch, RJ and Walker, CM (2000). Estimating erosion model erodibility parameters from media properties. Austr. J. of Soil Research 38, 265-84.
