# Supplementary material for: MINErosion 3: Using measurements on a tilting flume-rainfall simulator facility to predict erosion rates from post-mining landscapes in Central Queensland, Australia
Source: PLoS One. 2018 Mar 28;13(3):e0194230. doi: 10.1371/journal.pone.0194230 (PMC5874007; doi:10.1371/journal.pone.0194230)
Supplement: S2 File — (DOCX) [file pone.0194230.s002.docx]

**S2: Determination of the consolidation factor.**

**Objective:** To determine the effect of consolidation (increase in soil bulk density and strength) on soil erosion rates.

**Facilities:** The study was conducted in the field using a 4m long portable oscillating rainfall simulator similar in design and size to the laboratory simulator (Fig 1). It was based on the design of Bubenzer and Meyer (1965) and modified by Loch (1989)


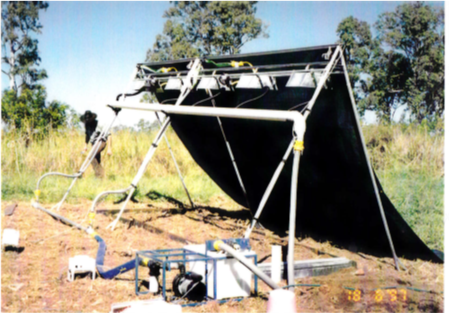

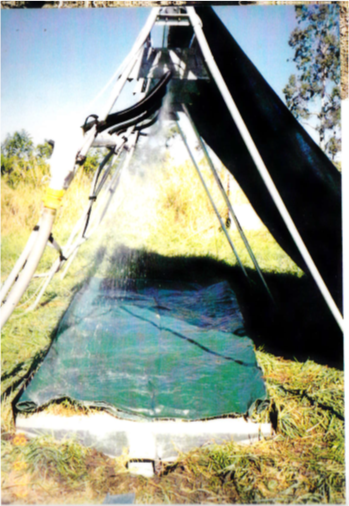


**Fig 1: The 4 m long field rainfall simulator. On the left is the complete set-up except the large 1000 l water tank is not shown. The shade cloth is used to reduce the effect of wind on the raindrop trajectories. The photo on the right shows the oscillating nozzle spraying water on the plot (4 m x 1.5m) which was covered with plastic for calibration of the rainfall intensity before and after each series of simulations.**

The 4 m simulator consist of two 2m sections joined together. There were 4 fan spraying nozzles (Spraying Systems VeeJet 80100 nozzles) at 1 m distances, located at 2.4 m above ground level. Spray patterns overlap and produced uniform distribution over a plot of 4m x 1.5m. Rainfall pattern is intermittent and intensity is adjustable. In the field it is usually set at 100 mm/h for 30 mins, a one in 20 year event for tropical rainstorm in Northern Queensland.

**Site preparation:**

**Blair Athol Mine**: 4 sites were selected on each of two soil types, a Black Earth (heavy clay 57% C,14% Si,28% S) and a Yellow Podzolic (sandy loam 24% C, 15% Si, 61% S ), covering 3 ages of rehabilitation (0, 2-4 years and 14 years) and an unmined site (nominally assumed as 30 years of rehabilitation and consolidation. All sites were on low slopes of 3-8 % (Black Earth) and 4-8 % (Yellow Podzolic). Vegetation cover were all estimated as above 50 %.

**Mount Isa Mines**: Sites were selected on 2 rehabilitation areas of 3 (Drill Pad) and 7 years (Spring Creek) and an unmined site. The soil was a rocky red duplex loamy soil with 4-10 % C, 5-10 % Si, 11-40 % S and 45–82 % rock and gravel. Slopes selected were 10 % and 20 % on the 3 year rehabilitation sites, and 15 % on the 7 year rehabilitation site.

From each site at Blair Athol and Mt Isa Mines, bulk samples were collected from the surface soil (representing fresh samples at 0 year of rehabilitation) and forwarded to the University of Queensland for determination of slope effects on the tilting flume-rainfall simulator facility in the Erosion Processes Laboratory (see S1 supplement). For each flume, three 200 l drum of bulk sample is required.

**Field Rainfall Simulations** :

At each site, two replicate plots of 4m x 1.5 m were prepared, and a supply of good quality water ( EC <200ds/l) is organised. Rainfall was applied at 100 mm/h for 30 mins. Run-off and sediment concentration were collected and timed at regular intervals during the simulations. Steady state rates of run-off and soil loss were measured. Infiltration rates were calculated.

Rainfall simulation were repeated after all the vegetation were removed to determine the soils inter-rill erodibility. This was followed with a series of overland flow simulation to determine the soils rill erodibility. Using these as inputs into MINErosion 3, the combined K_MUSLE_ were calculated. The effect of consolidation associated with age of rehabilitation is expressed as the relative K_M_ or the ratio of K_M_ over K_M_ of freshly laid material as shown in the Table 1.

**Table 1: The erodibility and relative erodibility of the various rehabilitated sites on Blair Athol and Mt Isa Mines. Measurements on the Black Earth at 2 years and unmined (30 years) did not result in adequate run-off to calculate K_M_ . *Measurement of the BE plot at 0 year could not be completed due to rain. K_M_ was estimated from the laboratory measurements.**

| **Mine** | **Site** | **Age rehab (year)** | **Soil type** | **% Clay** | **K_MUSLE_ (th/MJmm)** | **Relative K_M_** |
| --- | --- | --- | --- | --- | --- | --- |
| .Blair Athol |  | 0 | Yellow Podzolic | 24 | 0.015822 | 1.0 |
|  |  | 4 | Yellow Podzolic | 24 | 0.005532 | 0.35 |
|  |  | 15 | Yellow Podzolic | 24 | 0.001858 | 0.12 |
|  |  | 30 | Y Pod Unmined | 24 | 0.00149 | 0.09 |
|  |  | 0* | Black Earth | 57 | 0.0374 | 1.0 |
|  |  | 2 | Black Earth | 57 | NA | - |
|  |  | 15 | Black Earth | 57 | 0.001233 | 0.03 |
|  |  | 30 | B E unmined | 57 | NA | - |
| Mount Isa | Spring Crk | 0 | Red Podzolic | 18 | 0.07 | 1.0 |
|  |  | 7 | Red Podzolic | 18 | 0.07 | 0.13 |
|  |  | 30 | Red Podzolic | 14 | 0.03 | 0.07 |
|  | Drill Pad | 0 | Red Podzolic | 14 | 0.01 | 1.0 |
|  |  | 3 | Red Podzolic | 22 | 0.003 | 0.50 |
|  |  | 30 | Red Podzolic | 14 |  | 0.08 |

The data from Table 1 is plotted in Fig 2 and an exponential relationship was calculated for all the data. The regression for the pooled data is Y= 0.09 + 0.91(exp -0.4 X) with an R^2^ of 0.996

**Fig 2: The consolidation effect on soil erodibility expressed as Relative Erodibility against age of rehabilitation (years).**

The three soils with clay contents ranging from 14 % to 57 % fits well into a single relationship. Therefore, it can be assumed that other soils will follow this same relationship. This consolidation effect will be used as input into the MINErosion 3 model.

**References:**

1. Bubenzer, G.D.and Mayer, L.D. (1965). Simulation of rainfall and soils for laboratory research. Trans. Am. Soc. Agric. Eng 17: 1033-7
2. Loch, R.J. (1989). Aggregate breakdown under rain: its measurements and interpretation. PhD thesis, University of New England, Armidale, NSW. 139 pp;
3. Horn, CP; Yatapanage, K; So, HB and Mulligan, DR (2001). Erosion from rehabilitated and unmined sites at Blair Athol Coal Mine. Final Report to Blair Athol Mine, Centre for Mined Land Rehabilitation at The University of Queensland.
4. Horn, CP;Kopittke, GR; Williams, S; Yatapanage, K; So, HB and Mulligan, DR (1999). Erosion from Rehabilitated and Unmined Sites at Mount Isa Mine. Final Report to Mount Isa Mines Ltd, Centre for Mined Land Rehabilitation at The University of Queensland.
