# Supplementary material for: MINErosion 3: Using measurements on a tilting flume-rainfall simulator facility to predict erosion rates from post-mining landscapes in Central Queensland, Australia
Source: PLoS One. 2018 Mar 28;13(3):e0194230. doi: 10.1371/journal.pone.0194230 (PMC5874007; doi:10.1371/journal.pone.0194230)
Supplement: S3 File — (DOCX) [file pone.0194230.s003.docx]

**S3.** **Procedures used for validation of annual erosion rates and individual rainstorm events.**

**Mean annual erosion rates estimation using RUSLE:**

1. For the selected location, the R factor (annual EI_30_ values) was calculated from long-term weather data
2. In MINErosion 3, the soil or spoil for the selected mines were selected from the embedded database. This will already have information on K_MUSLE_ , infiltration rate and rock cover. They were derived from measurements conducted on the laboratory tilting flume-rainfall simulator facility.
3. Select L and S of the field plot of interest. MINErosion 3 computes the LS factor using the RUSLE procedure.
4. Select the period of consolidation in years to compute the effective K_MUSLE_
5. Select the % vegetation cover (C) reported for the field plot, while the P factor is assumed as 1.
6. Compute the average annual soil erosion rate A using the RUSLE equation.
7. Repeat steps a to f for the different field plots.
8. A linear regression was conducted between the calculated/predicted A against measured A (average of total annual erosion over the period of observation). A Nash-Sutcliffe analysis was also conducted on this data.

**Individual rainstorm events estimation using MUSLE:**

1. The EI_30_ value for each recorded storm event was computed.
2. As MINErosion 3 used 100 mm.hr^-1^ as the default rainstorm, the effective storm duration D was determined that give the same EI_30_ value as the observed rainstorm. Q and q_p_ are computed according to equations (1) and (11).
3. Select soil or spoil for the Curragh mine from the embedded database and MINErosion 3 will compute K_MUSLE_ for bare soil/spoil.
4. Select L and S of the field plot and MINErosion 3 will compute the LS factor using the RUSLE manual
5. Select the period of consolidation (years) of the plot of interest and the effective K_MUSLE_ will be computed.
6. Select the % vegetation cover (C) reported for the field plot, while the P factor is assumed as 1
7. Compute the storm event erosion rate A.
8. Repeat steps a) to g) for all recorded storm events.
9. A linear regression was conducted between predicted and measured A, and a Nash-Sutcliffe analysis conducted on this data.
